# Modulating signalling lifetime to optimise a prototypical animal opsin for optogenetic applications

**DOI:** 10.1007/s00424-023-02879-9

**Published:** 2023-12-01

**Authors:** Jessica Rodgers, Phillip Wright, Edward R. Ballister, Rebecca B. Hughes, Riccardo Storchi, Jonathan Wynne, Franck P. Martial, Robert J. Lucas

**Affiliations:** 1 Centre for Biological Timing, Division of Neuroscience, Faculty of Biology, Medicine and Health, University of Manchester, Manchester, M13 9PT UK; 2 Department of Biomedical Engineering, Columbia University, New York, NY 10027 USA; 3 Department of Microbiology and Immunology, Vagelos College of Physicians and Surgeons of Columbia University, New York, 10032 NY USA

**Keywords:** Optogenetics, Opsin, Rhodopsin, Arrestin, G-protein, GPCR, Kinetics, Deactivation

## Abstract

Animal opsins are light activated G-protein-coupled receptors, capable of optogenetic control of G-protein signalling for research or therapeutic applications. Animal opsins offer excellent photosensitivity, but their temporal resolution can be limited by long photoresponse duration when expressed outside their native cellular environment. Here, we explore methods for addressing this limitation for a prototypical animal opsin (human rod opsin) in HEK293T cells. We find that the application of the canonical rhodopsin kinase (GRK1)/visual arrestin signal termination mechanism to this problem is complicated by a generalised suppressive effect of GRK1 expression. This attenuation can be overcome using phosphorylation-independent mutants of arrestin, especially when these are tethered to the opsin protein. We further show that point mutations targeting the Schiff base stability of the opsin can also reduce signalling lifetime. Finally, we apply one such mutation (E122Q) to improve the temporal fidelity of restored visual responses following ectopic opsin expression in the inner retina of a mouse model of retinal degeneration (*rd1*). Our results reveal that these two strategies (targeting either arrestin binding or Schiff-base hydrolysis) can produce more time-delimited opsin signalling under heterologous expression and establish the potential of this approach to improve optogenetic performance.

**Supplementary Information:**

The online version contains supplementary material available at 10.1007/s00424-023-02879-9.

## Introduction

Optogenetics is the process of controlling the activity of cells using light by ectopic expression of light sensitive proteins. It has proved a crucial development for both basic and translational research, allowing precise temporal and spatial control of a wide variety of cellular activities. The most widely used class of photosensitive proteins for optogenetics are the retinaldehyde-binding opsins. There are two main classes of opsins: type I microbial opsins (light-sensitive ion channels) and type II animal opsins (light-sensitive G-protein-coupled receptors) [1, 19, 26, 34]. While both opsin classes have been used for basic research [15, 43, 52] and more translational applications [9, 11, 13, 17, 20, 23, 24, 39, 42, 53, 60, 61, 66, 71], here we consider the latter group, animal opsins, which has several particularly useful characteristics as optogenetic actuators. Most importantly, they represent an opportunity to target a class of cellular process (G-protein signalling cascades) with wide physiological and disease relevance. In addition, the signal amplification inherent in many G-protein signalling cascades allows them to attain high photosensitivity compared to most other optogenetic tools [4]. Finally, the human genome contains several members of the type II opsin group, raising the attractive possibility of using native human proteins for therapeutic applications [9, 11, 23, 24, 66, 71], including vision restoration, control of blood glucose, and muscle contraction.

One inherent challenge of using animal opsins in optogenetics is their tendency to drive relatively long-lasting light responses (often lasting many tens of seconds) when expressed outside of their natural environment. This property constrains one of the key advantages of optogenetics, achieving cellular control with high spatial and temporal resolution. It is a particular concern for the most promising clinical application of this technology, restoring photosensitivity in retinal degeneration, for which spatiotemporal acuity is a critical determinant of visual performance. Other applications of animal opsins which require precise temporal control, such manipulating heart rate or intestinal contraction [66], would also be enhanced by more time-delimited responses to light.

Native photoreceptors employing animal opsins display excellent temporal resolution. This capacity is achieved in part by rapidly quenching the signalling of photoactivated opsins in a two-step process: First, the activated opsin is phosphorylated by a G-protein-coupled receptor kinase (GRK), which partially reduces signalling activity and allows binding by arrestin, which completely quenches signalling. This mode of deactivation requires both a suitable arrestin and G-protein receptor kinase, for example, GRK1 and arrestin-1 in the case of rod opsin [28, 37, 68]. This form of receptor desensitisation is not unique to opsins and is found in many GPCR signalling pathways, including beta-2 adrenergic receptors, which bind GRK2 and Beta-arrestin2 [7, 8, 36]. While GRK2/3/5/6 and Beta-arrestin1/2 are ubiquitously expressed, the opsin-specific GRK1/2 and arrestin-1/4 are found in rod and cone photoreceptors only. As a result, in many optogenetic applications, the necessary receptor kinase and arrestin may be absent in the host cell. Some opsins (including human visual opsins such as rod and cone opsin) also have an intrinsic partial deactivation mechanism in the form of hydrolysis of the Schiff base linkage between the agonist, all-*trans* form of retinal, and opsin apoprotein. This leads to decay of the signalling active ‘meta II’ state of the opsin [6, 29, 65]. In theory, both Schiff-base hydrolysis and arrestin binding could be targeted to reduce the lifetime of photoactivated animal opsins in optogenetic applications.

Here, we tested these approaches for improving animal opsin optogenetics. We used a prototypical animal opsin, human rod opsin. Rod opsin is the most extensively characterised of all opsins, and its deactivation by both kinase/arrestin and Schiff-base hydrolysis mechanisms is well established. Human rod opsin is also a potentially important optogenetic tool for experimental and therapeutic applications in which spatiotemporal resolution is essential; it is a human protein that expresses well ectopically, is highly sensitive, and capable of coupling to native Gi/o/t pathways [2, 5, 70]. Rod opsin has been successfully expressed in the surviving retinal cells of retinally degenerate *rd1* mice to restore basic image-forming vision at physiological light intensities [11, 23]. Its main disadvantage, as revealed by those *in vivo* studies, appears to be the slow latency and decay of light responses observed in treated blind animals compared to visually intact wildtype controls, which is likely to curtail the spatiotemporal acuity of restored vision and other translational applications.

We apply a live cell bioluminescence resonance energy transfer (BRET)–based readout of G-protein activation [44, 45] to show that either enhancing arrestin binding or accelerating Schiff base hydrolysis can reduce the lifetime of the rod opsin photoresponse under heterologous expression. We finally confirm the potential of such approaches to improve temporal resolution in an optogenetic application by comparing electrophysiological responses from degenerate *rd1* retinas treated with unmodified human rod opsin or bearing a single amino acid substitution (E122Q) targeting Schiff base hydrolysis.

## Results

### A live cell assay of G-protein activation to measure the kinetics of opsin signalling

**Fig. 1:**
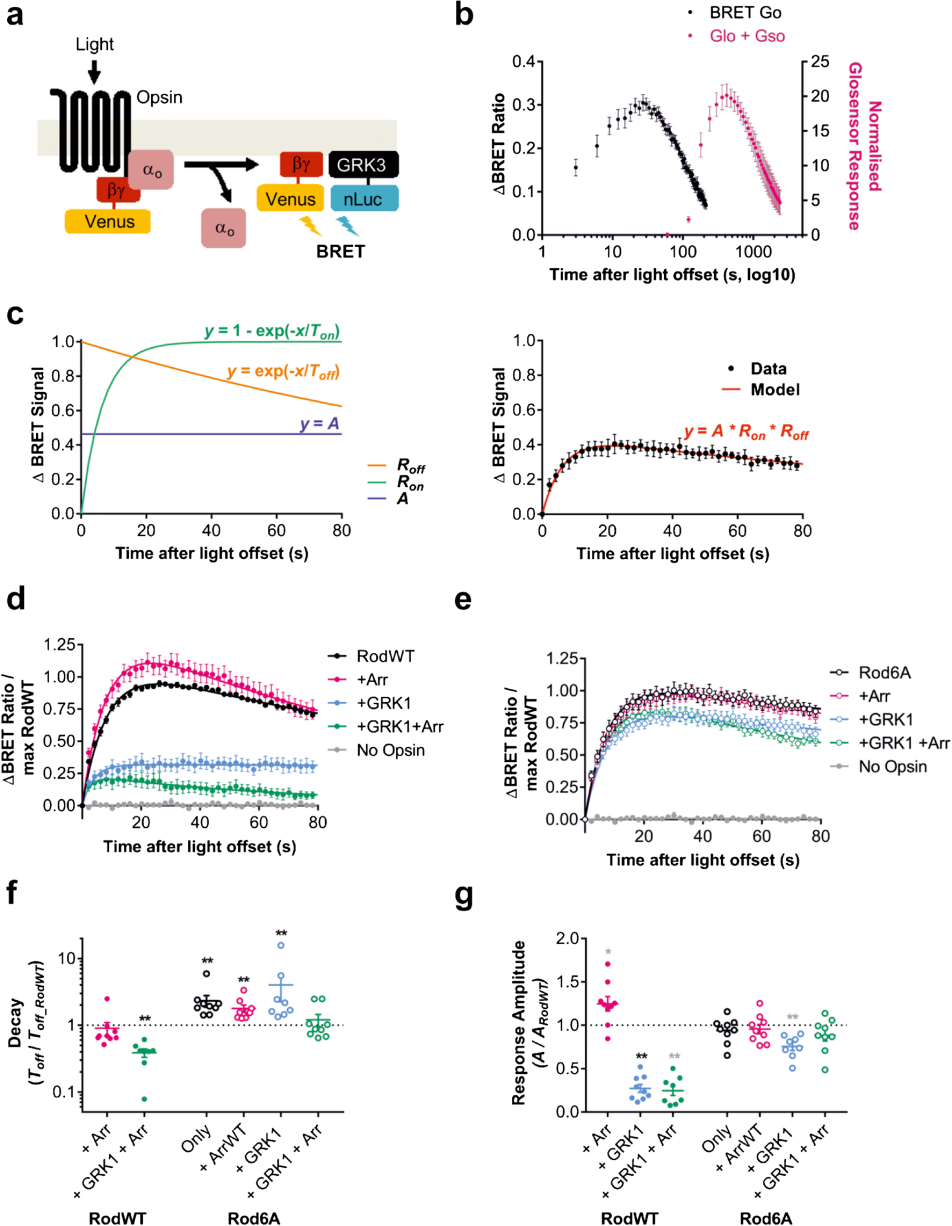
**a** Ratiometric BRET assay of G-protein activation. Dissociation of the G-protein heterotrimer is detected when BRET occurs between free Gβγ-dimer tagged with yellow fluorescent protein (Venus) and membrane-localised GRK3-fragment fused to nanoluciferase (which continuously emits light at 470 nm), leading to emission of 535 nm light. As G-protein heterotrimer reassociates, the binding site for GRK3 fragment is blocked by Gα subunit and emission of 535 nm fluorescent light decreases. BRET ratio is calculated as light emitted at 535 nm/light emitted at 470 nm. **b** Temporal resolution of rod-opsin driven light responses with BRET assay of G-protein activation (BRET Go: black) is faster than for secondary messenger Glosensor Gso assay (Glo + Gso: Pink). **c** Baseline normalised BRET responses are modelled using a simple dual exponential model (left panel) consisting of a scaling factor (*A*), a one-phase exponential association curve (*R*_*on*_), and one-phase exponential decay curve (*R*_*off*_). The three parameters that define this model, *A, T*_*on*_, and *T*_*off*_*,* are adjusted to fit model to data using nonlinear regression (right panel). The best fit parameter *A* is used as a measure of response amplitude, and *T*_*off*_ is used as a measure of response decay rate. **d**–**e** Time course of BRET response to 1 s 485 nm light (16.5 log photons) normalised to pre-flash baseline (= 0) and maximum response from wildtype Rod opsin positive control (= 1). Responses show **d** wildtype Rod opsin (RodWT – filled makers) or** e** phosphonull Rod opsin 6A mutant (Rod6A – unfilled markers) in presence of either G-protein receptor kinase 1 (GRK1) and/or visual arrestin (Arr). **f** Relative response decay, measured as best fit *T*_*off*_ (s) for each condition divided by best fit *T*_*off*_ for rod opsin positive control (*T*_*off_RodWT*_). **g** Relative response amplitude, measured as best fit scaling factor (*A*) for each condition divided by best fit scaling factor for rod opsin positive control (*A*_*Rod_WT*_). Glosensor and BRET data shown in **b** and **c** are mean ± standard error of mean for *n* = 3 replicates. BRET data shown in **d**–**g** are mean ± standard error of mean for *n* = 8–9 replicates from 3 separate transfections. For statistical analysis, a two-tailed one-sample Wilcoxon signed ranks test was used to compare each condition relative to Rod control (theoretical median = 1). **p < 0.01; *p < 0.05; not significant where no asterisk is displayed. Grey asterisk = significant for uncorrected alpha (0.05), black asterisk = significant for Sidak corrected alpha (0.007 for *A*, 0.009 for *T*_*off*_)

We first used a live cell assay capable of revealing opsin signalling with good temporal fidelity, by using a BRET-based reporter of G-protein activation [44, 45]. Briefly, we transiently transfected HEK293T cells with human rod opsin, Gα_o_, split Venus-tagged Gβγ subunits (sVβγ), and a nanoluciferase-tagged GRK3 fragment (nLuc-GRK3, Fig. 1a). In this system, light-dependent rod opsin-driven dissociation of the Gα_o_βγ heterotrimer results in BRET between the liberated Gβγ and nLuc-GRK3. Accordingly, a light flash drove an increase in the ratio of light emitted by Venus to that emitted by nanoluciferase (BRET ratio) in these cells. Rod-opsin driven light responses have previously been detected in live cells using Glosensor, a luminescent reporter for the second messenger cAMP that is impacted by Gα_i/o/t_ pathways [2, 3]. In a side-by-side comparison, we found the BRET signal both rose and fell much faster than the Glosensor response (Fig. 1b), consistent with its ability to report an earlier stage in the signalling cascade (G-protein activation) and demonstrating its superiority for reporting the kinetics of opsin photoresponse.

To facilitate quantitative analysis of the rod opsin response kinetics, we described the BRET response profile using a simple 3-parameter model (Fig. 1c, left panel) which we fit using nonlinear regression (Fig. 1c, right panel). This model consisted of an exponential association curve for the response onset (*R*_*on*_); an exponential decay curve for the response decay (*R*_*off*_); and a scaling factor representing a measure of response amplitude (*A*). The three parameters that define the components of this model are *T*_*on*_,* T*_*off*_, and *A*. *T*_*on*_ is defined by the rate of accumulation of the BRET signal which, as opsin activation by light is effectively instantaneous over this timescale, will be primarily defined by the latency of the BRET assay response. *T*_*off*_ is defined by the rate of decay of the BRET signal and *a priori* is expected to be influenced both by the rate of opsin deactivation and rate at which BRET signal recovers to baseline in the absence of further G-protein activation. *A* reflects the peak amplitude of the BRET response. *T*_*on*_ and *T*_*off*_ are in units of seconds, and *A* is a dimensionless scaling factor. To meet our objective of enhancing temporal resolution, we aimed to identify interventions that minimised the lifetime of photoactivated opsin (reflected in reduced *T*_*off*_). As *A* is a product of free G-protein accumulation over time, reductions in *T*_*off*_ have the potential to alter *A*. The converse, where *T*_*off*_ is affected by *A,* is also possible in principle, and would be a greater concern as it would compromise our ability to use this assay to compare response kinetics. To determine whether this was the case, we applied variations in flash intensity to produce a range of response amplitudes and found that in fact *A* and *T*_*off*_ were not significantly correlated (Supplementary Fig. 1). However, we did find a significant inverse correlation between *T*_*on*_ and *T*_*off*_*,* suggesting that the OFF component of our model may partially contaminate the ON component, or vice versa.

### Quenching rod opsin responses using visual arrestin

We first applied this method to determine the effect of introducing the components for rod opsin deactivation that are normally present in rod photoreceptors. Co-expression of visual arrestin (Arr) with rod opsin (Fig. 1d) was associated with a modest increase in response amplitude compared to rod opsin alone and no significant change in *T*_*off*_ (Fig. 1f; *T*_*off*_ = 166.16 s ± 10.06 for RodWT vs 161.45 s ± 48.98 for Rod + Arr; mean ± SEM, all comparisons use one-sample Wilcoxon signed rank test, unless stated otherwise, *p* = 0.129). The additional inclusion of rod opsin’s native G-protein kinase (GRK1) with Arr (Fig. 1d and g) had the expected effect on longevity, with *T*_*off*_ reduced to 39% of rod opsin alone (*T*_*off*_ = 65.57 s ± 10.54 for RodWT + Arr + GRK1; *p* < 0.01). There was an additional effect on amplitude (*A,* Fig. 1g), which was reduced from 0.57 ± 0.04 for RodWT to 0.13 ± 0.02 for RodWT + Arr + GRK1 (*p* < 0.01). Interestingly, introduction of GRK1 without Arr also had dramatic effects on the rod opsin driven response (Fig. 1d). Thus, compared to rod opsin alone, inclusion of GRK1 alone dramatically reduced amplitude (*A* = 0.15 ± 0.02 for RodWT + GRK1, *p* < 0.01) and rendered the light response sufficiently long lived that T_off_ was essentially infinite.

Signal termination by GRK1 and arrestin is thought to originate with phosphorylation of residues on the rod opsin C-terminal tail. To determine whether this mechanism explained the effects of GRK1 and arrestin in this assay, we generated a version of rod opsin (Rod6A) lacking these phosphorylation sites. Light response driven by Rod6A (Fig. 1e) had a similar amplitude from that of the native rod opsin (RodWT, Fig. 1g), but was more long lasting (*T*_*off*_ = 389.14 s ± 88.3 for Rod6A, *p* = 0.004) consistent with the disruption of possible interactions between opsin and native kinases/arrestins in HEk293 cells (see Supplementary Fig. 2 for more detailed comparison between RodWT and 6A). Compared to RodWT, *T*_*off*_ was still significantly slower in Rod6A after addition of arrestin alone (*T*_*off*_ = 301.04 s ± 48.2 for Rod6A + Arr, *p* < 0.01), but not when both arrestin and GRK1 were included (*T*_*off*_ = 213.85 s ± 55.4 for Rod6A + Arr + GRK1, *p* = 0.820), suggesting that Rod6A has partially, but not completely, lost this mechanism of signal termination. The dramatic effects of GRK1 alone on the RodWT response were largely lost for Rod6A, with amplitude moderately reduced compared to RodWT (*A* = 0.42 ± 0.03 for Rod6A + GRK1).

### Phosphorylation-independent mutants of arrestin

The experiments with Arr and GRK1 confirm that this method of rod opsin signal termination can be functional in heterologous expression and can achieve reductions in *T*_*off*_. However, they also reveal arrestin-independent effects of GRK1, in the form of a substantial reduction in amplitude and, in the absence of Arr, an increase in *T*_*off*_. These unwanted effects of GRK1 are lost in Rod6A (suggesting they are produced by rod opsin phosphorylation), but so is the arrestin-dependent reduction in *T*_*off*_*.* To resolve this, we turned to phosphorylation-independent arrestin mutants. Initially developed as a treatment for Oguchi disease [27], which occurs in individuals with rod opsin and GRK1 mutations that prevent phosphorylation, these arrestin mutants have their ‘phosphorylation sensor’ removed, increasing their affinity for photoactivated unphosphorylated opsin. We reasoned that if these were able to improve temporal resolution of rod opsin, they would remove the requirement for GRK1 phosphorylation and potentially allow responses with the desired larger amplitude and shorter duration.

**Fig. 2:**
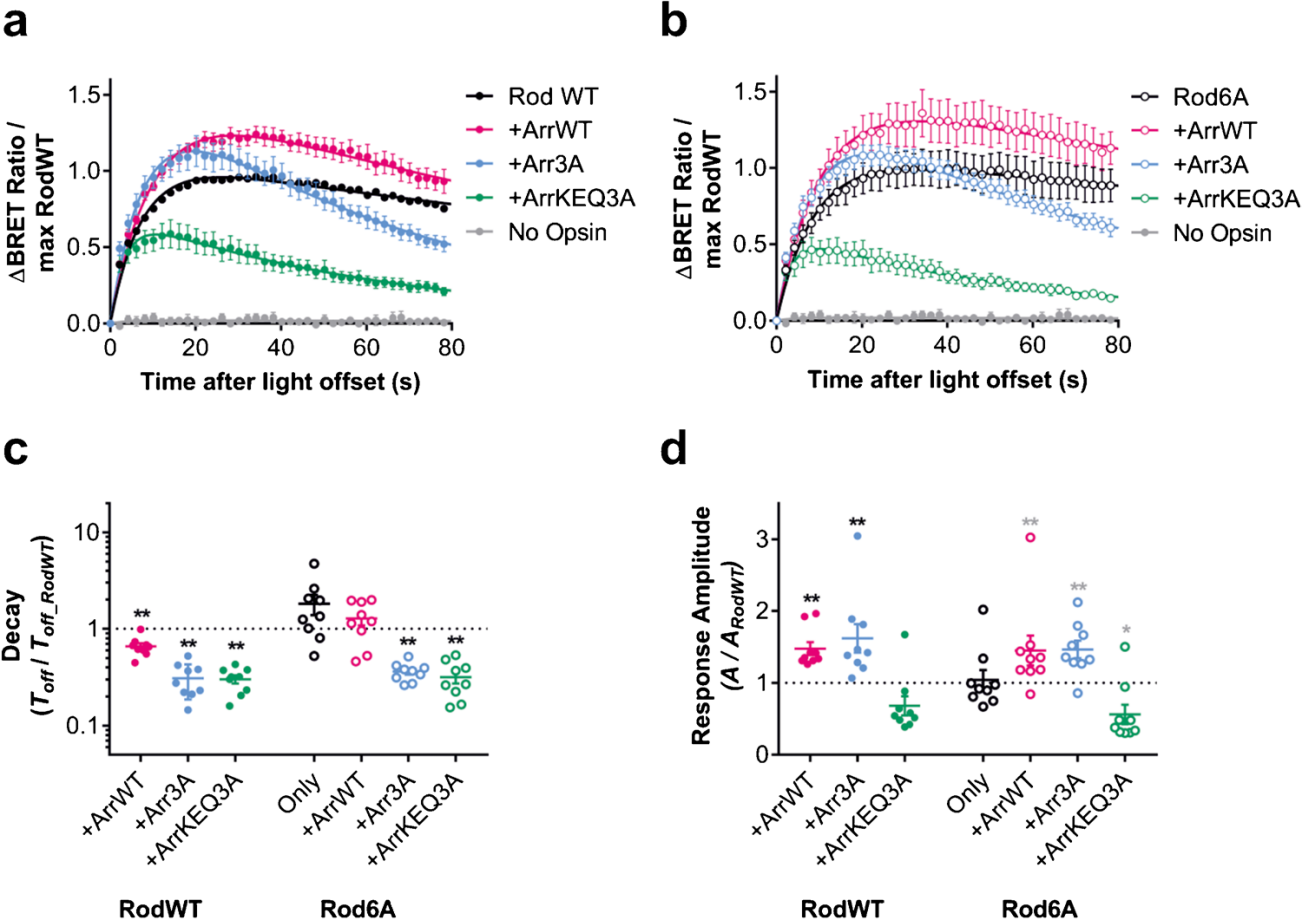
**a**–**b** Time course of BRET light responses for **a** wildtype Rod opsin (RodWT) and **b** phosphonull Rod opsin 6A mutant (Rod6A) when co-transfected with either wildtype visual arrestin (ArrWT) or arrestin mutants with intermediate (Arr3A) or strong affinity (ArrKEQ3A) for unphosphorylated active rod opsin. Responses are to 1 s 485 nm light (16.5 log photons). Data are normalised to pre-flash baseline (= 0) and maximum response of RodWT (= 1). **c** Response decay (measured as fold change in best fit *T*_*off*_, s) of both RodWT and Rod6A is decreased when arrestin mutants Arr3A and ArrKEQ3A are added. **d** Response amplitude (measured as fold change in best fit scaling factor *A*) of both RodWT and Rod6A is partially increased in presence of ArrWT and Arr3A, but not ArrKEQ3A. Data are mean ± standard error of mean of *n* = 9 replicates from 3 separate transfections. In **c**–**d,** a two-tailed one-sample Wilcoxon signed ranks test was used to compare each condition relative to Rod control (theoretical median = 1). *p < 0.05; **p < 0.01; not significant where no asterisk is displayed. Grey asterisk = significant for uncorrected alpha (0.05), black asterisk = significant for Sidak corrected alpha (0.007 for *A* and *T*_*off*_)

We tested Rod opsin co-transfected with wildtype arrestin (ArrWT) or one of two arrestin mutants, Arr3A and ArrKEQ3A. These arrestin mutants have previously shown to have intermediate and high affinity for purified unphosphorylated rod opsin protein, respectively [64] (Fig. 2a). The high affinity arrestin KEQ3A mutant reportedly has similar affinity for active unphosphorylated rod opsin (R*) as wildtype arrestin has for phosphorylated active rod opsin (P-R*). While the Arr3A mutant has previously been show to accelerate rod opsin deactivation in rod photoreceptors [55], the impact of ArrKEQ3A on G-protein signalling dynamics is unknown. We also tested the arrestin mutants with phosphonull Rod6A to confirm any effects were truly phosphorylation-independent (Fig. 2b).

Both phospho-independent arrestins shortened the lifetime of RodWT by about 70% (Fig. 2c; *T*_*off*_ = 240.64 s ± 24.13 for RodWT to 69.07 s ± 8.03 for RodWT + Arr3A, and 68.0s ± 5.03 for RodWT + ArrKEQ3A), compared to 34% reduction produced by ArrWT alone (Fig. 2c; *T*_*off*_ = 160.19 s ± 21.2 for RodWT + ArrWT)**.** As expected, this effect was retained when RodWT was replaced by Rod6A (*T*_*off*_ = 83.79 s ± 5.89 for Rod6A + Arr3A and 74.41 s ± 12.1 for Rod6A + ArrKEQ3A) with *T*_*off*_ reduced by 64% and 68% compared to RodWT, for Arr3A and ArrKEQ3A respectively. There was a notable difference between the two arrestin mutants in response amplitude (Fig. 2d). While ArrKEQ3A suppressed response amplitude to approximately half that of RodWT, Arr3A increased response amplitude by ~ 1.5-fold for both RodWT and Rod6A (*A* = 0.50 ± 0.07 for RodWT, 0.29 ± 0.03 for RodWT + ArrKEQ3A, 0.22 ± 0.01 for Rod6A + ArrKEQ3A, 0.76 ± 0.09 for RodWT + Arr3A, 0.69 ± 0.08 for Rod6A + ArrKEQ3A; *p* = 0.129, 0.012, 0, and 0.012, respectively).

### Improving efficiency of opsin deactivation using opsin-arrestin fusions

**Fig. 3:**
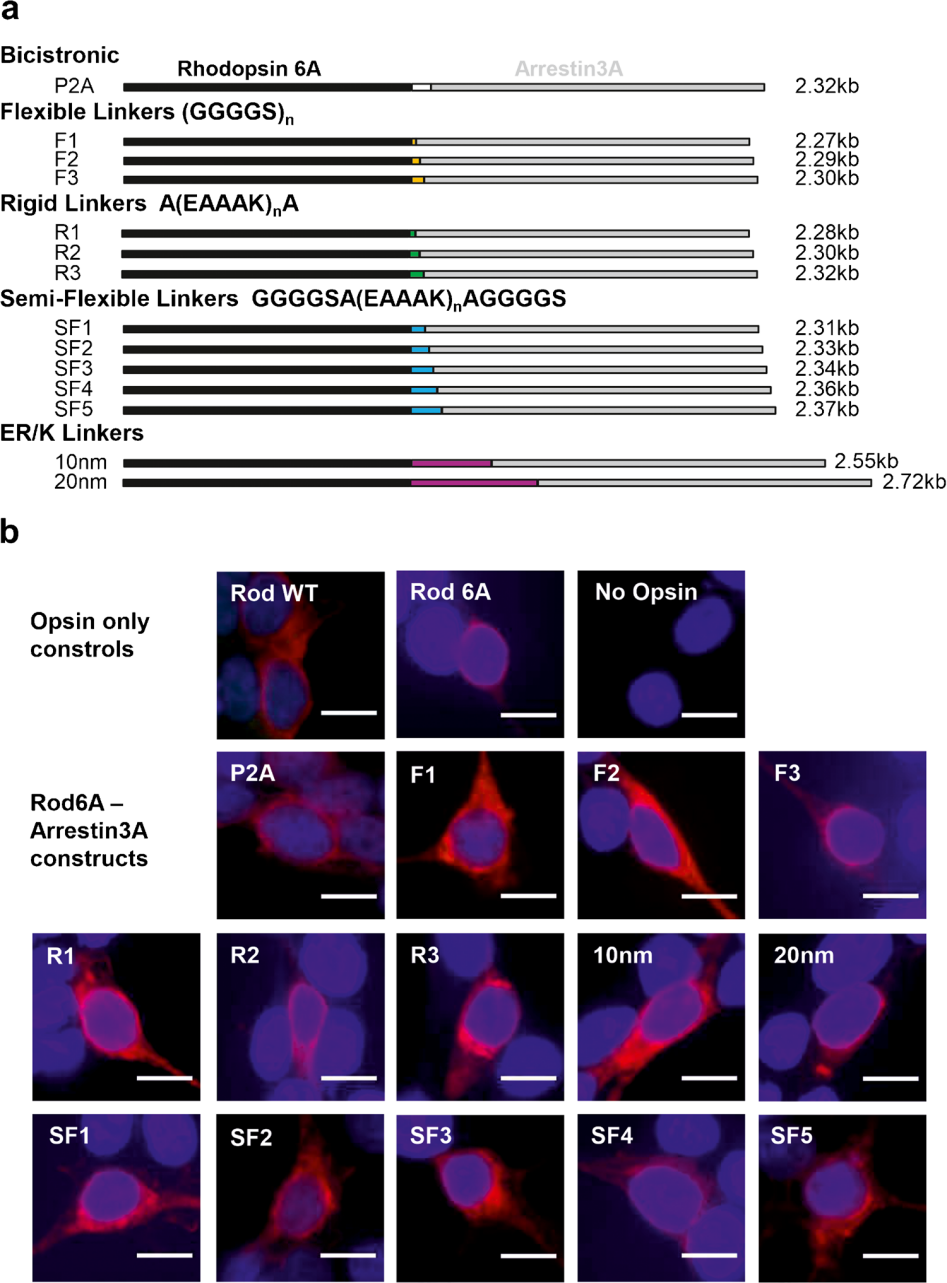
**a** Diagram of Rod opsin-Arrestin 3A bicistronic and fusion constructs. Linkers with different biophysical properties and lengths were used to produce 13 fusion constructs. Length of rod opsin, arrestin, and linkers are to scale, with construct size in kilo base pairs shown on right. **b** Heterologous expression of wildtype Rod opsin (RWT) only, phosphonull Rod opsin 6A only, Rod opsin6A co-expressed with Arrestin 3A (R6-P2A-Arr3A), or Rod opsin 6A tethered to Arrestin 3A by linker in HEK293T cells labelled with anti-rhodopsin 4D2 antibody (red) and DAPI nuclear stain (blue). Scale bar = 10 μm

The combination of Rod6A and Arr3A improved the temporal resolution of rod opsin, without affecting response amplitude. We next explored whether physically tethering these two proteins to create fusions may allow even more efficient deactivation. To this end, we designed a series of fusion constructs with a variety of linkers (Fig. 3a), to determine which allowed optimal interaction between Rod6A and Arr3A. These include flexible glycine-serine linkers with a high degree of rotational freedom [16]; rigid alpha-helix forming linkers, which limit interaction of the two proteins; semi-flexible linkers, consisting of a rigid linker with flexible ends [41]; and longer naturally-occurring ER/K linkers, which possess alternating charge, making the linker unlikely to interact with protein domains at either end [58]. We also varied the length of the different linkers, with frequency of interaction decreased as linker length increased.

Each Rod6A-Arr3A fusion was compared with co-expression of the two proteins without physical association. In an attempt to approach a 1:1 stoichiometric ratio, we switched from simply co-transfecting expression vectors for the two components (used to generate data in Fig. 2) to employing a single bicistronic vector (Rod6A-P2A-Arr3A) using the self-cleaving P2A sequence to produce the two proteins from a single open reading frame [33]. Immunostaining of the fusion constructs (Fig. 3b) confirmed that all fusions were expressed in Hek293T cells. Using fluorescent intensity of anti-rhodopsin 4D2 staining as a measure of expression level, we found that expression of fusion constructs was generally comparable to RodWT positive control (Supplementary Fig. 3a), except for F3, R2 and SF3, which were notably lower.

**Fig. 4:**
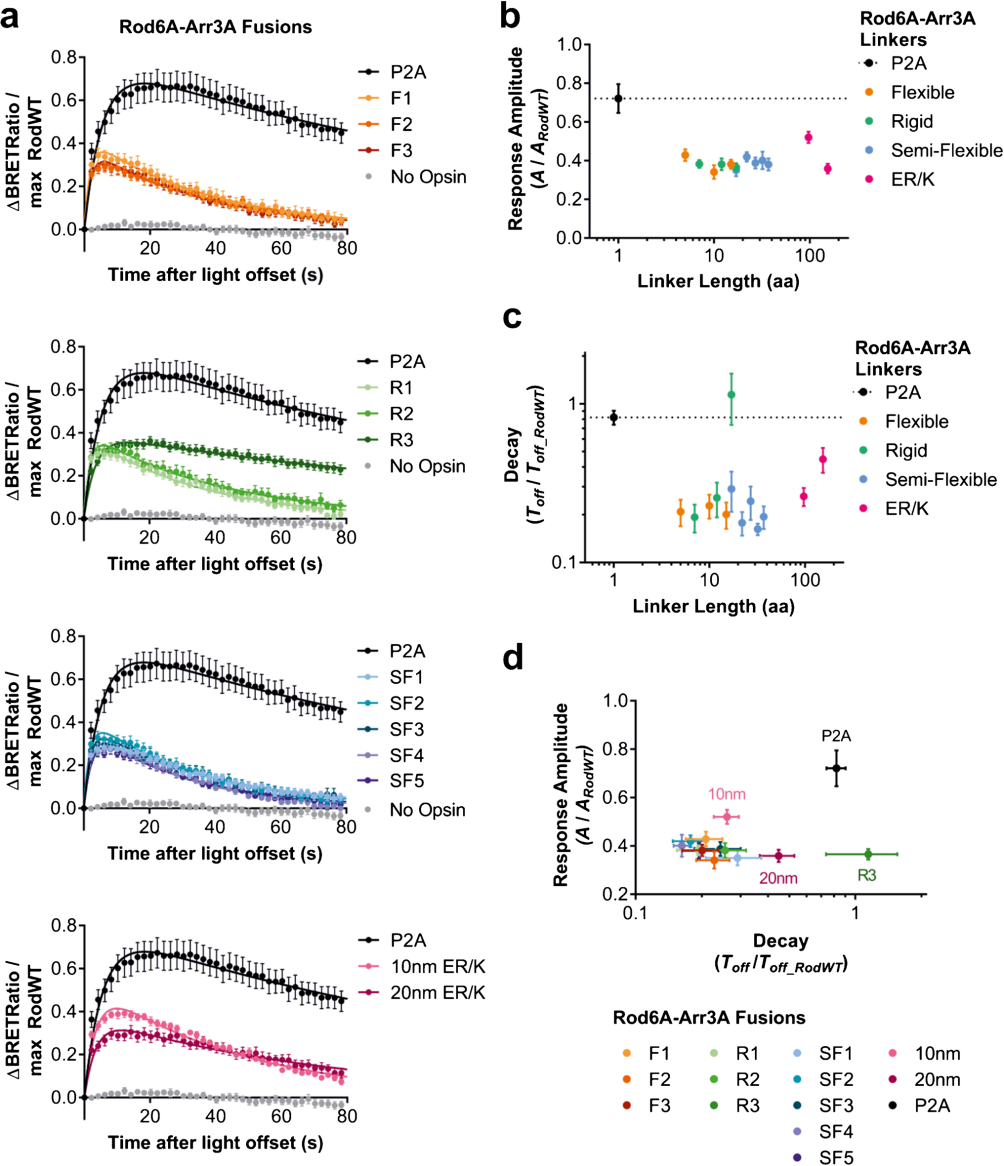
**a** Time course of BRET light responses for co-expression or fusions of phosphonull rod opsin mutant (Rod6A) and intermediate affinity arrestin mutant 3A (Arr3A). Responses are to 1 s 485 nm light (16.5 log photons). Data are normalised to pre-flash baseline (= 0) and maximum response of rod opsin positive control (= 1). The data for different linkers are shown in 4 panels for clarity, with the same P2A and no-opsin data plotted across the 4 panels for comparison. **b** Response amplitude (measured as fold change in best fit scaling factor *A* from Rod opsin positive control) is decreased for fusion constructs compared to co-expression with bicistronic P2A vector (dashed line), with no obvious relationship of linker length or composition with amplitude. **c** Response decay (measured as fold change in best fit *T*_*off*_, from Rod opsin positive control) is faster for most fusion constructs compared to co-expression with P2A (dashed line). Linker R3 was notably very slow to deactivate. **d** Comparing response amplitude and decay (measured as scaling factor *A* and *T*_*off*_, respectively) of Rod6A-Arr3A fusions shows that most linker lengths and compositions result in similar response properties. Of all fusion constructs, the ERK 10-nm linker provided the largest response amplitude while maintaining fast response decay. Data shown are mean ± standard error of mean of *n* = 10–13 replicates for P2A and fusions and *n* = 20 for RodWT from 4 separate transfections

All Rod6A-Arr3A fusion constructs retained the ability to drive light responses (Fig. 4a), albeit with response amplitude reduced to 40–60% of untethered co-expression (Fig. 4b). Turning to response lifetime, our first observation was that introducing Arr3A using the bicistronic vector had a smaller impact on *T*_*off*_ than previously observed in the co-transfection studies (Fig. 2). The origin of this is unclear; possible explanations include the enforced 1:1 stoichiometry or detrimental effects of the P2A peptide sequence, which remains on the opsin C-terminus. Tethering did produce a greater reduction in *T*_*off*_, with fusions showing response lifetimes by 48–81% shorter compared to co-expression of Rod6A and Arr3A. The only exception, Rod6A-R3-Arr3A, had a 20% longer response lifetime than the Rod6A-P2A-Arr3A construct.

Comparison of response parameters across the various fusion constructs revealed firstly that there was no strong relationship between response amplitude and *T*_*off*_ (Fig. 4d). The implication that these parameters are at least partially dissociable in this dataset suggests that our approach is suitable for identifying the fusion that provides the best combination of a light response with high signalling efficiency (large *A*) and short lifetime (low *T*_*off*_). Of all fusions, Rod6A-10 nm-Arr3A offered the best trade-off of speed (mean ± SEM, *T*_*off*_ = 47.44 s ± 3.88) and response amplitude (*A* = 0.17 ± 0.02). Performance as quantified in these terms did not systematically vary as a function of linker length or composition, as most constructs clustered around similar values for response amplitude and decay (Fig. 4d) with few outliers.

As a final test that the enhanced affinity of the Arr3A mutant for unphosphorylated opsin was a critical consideration in these fusion proteins, we collected a parallel dataset employing fusions of Rod6A with ArrWT (Supplementary Fig. 4). We found that although these fusions exhibited 68–90% faster decay (range = 123.1 s to 390.2 s) compared to co-expression of Rod6A and ArrWT (*T*_*off*_ = 1237.13 s ± 868.5), they were still notably slower than Rod6A-Arr3A fusions, such as Rod6A-10 nm-Arr3A. The slower decay demonstrated by ArrWT compared to Arr3A is consistent with our findings for co-transfection of Rod6A with ArrWT and Arr3A, shown in Fig. 2, suggesting that the increased affinity of Arr3A for R* contributes to the faster* T*_*off*_ observed in Rod6A-Arr3A fusions.

### Rod opsin mutants with faster meta-II decay have reduced light response lifetime

**Fig. 5:**
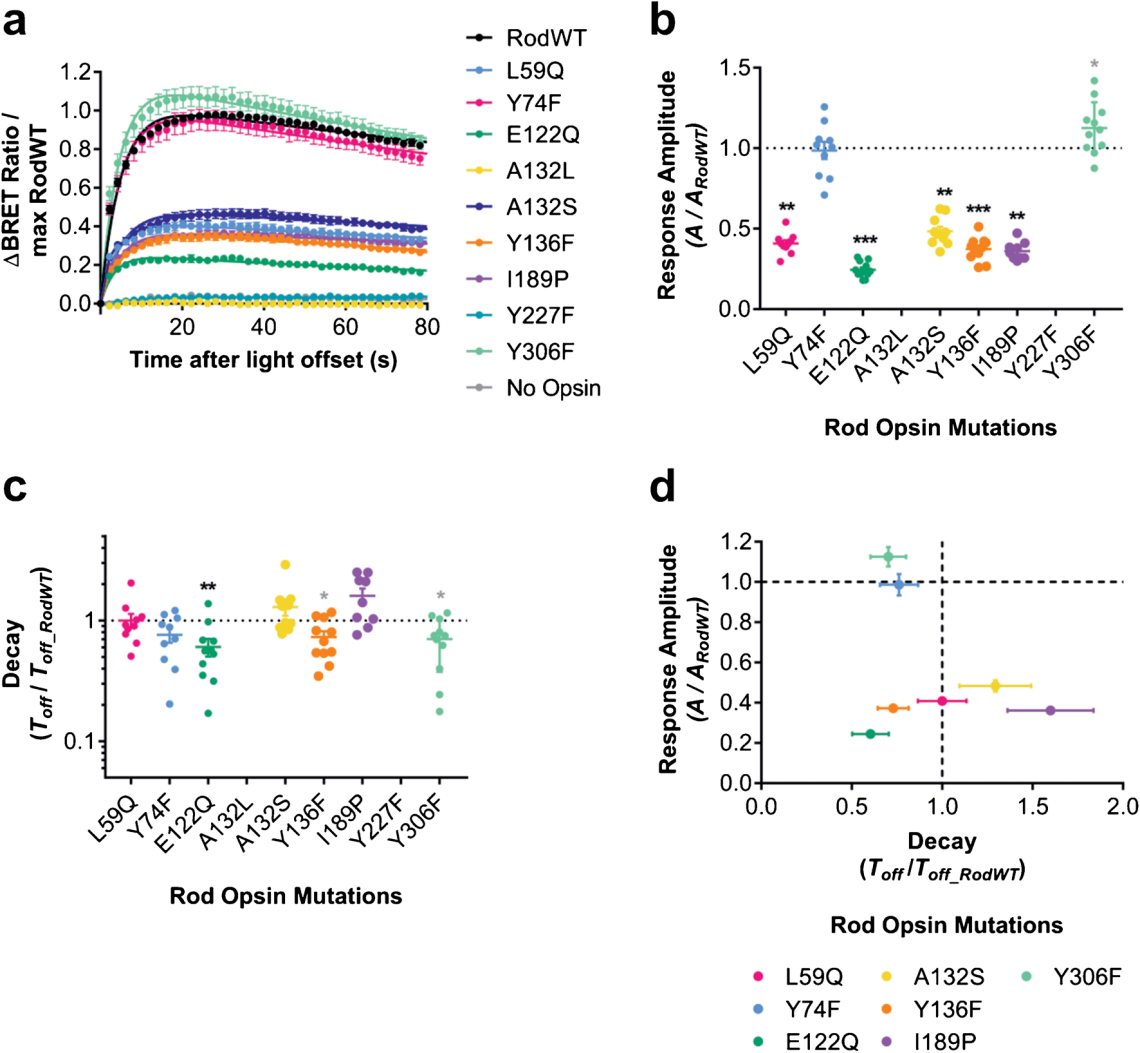
**a** Time course of BRET light responses for rod opsin wildtype (RodWT) and mutants reported to have faster meta-II decay. Responses are to 1 s 485 nm light (16.5 log photons). Data are normalised to pre-flash baseline (= 0) and maximum response of rod opsin positive control (= 1). **b** Response amplitude (measured as fold change in best fit scaling factor *A* from Rod opsin positive control) is generally smaller for meta-II decay mutants, compared to RodWT, although Y74F and Y306F have comparable and larger amplitudes respectively. **c** Response decay (measured as fold change in best fit *T*_*off*_, s from Rod opsin positive control) varies across rod opsin meta-II decay mutants, with E122Q, Y136F, and Y306F showing faster responses and L59Q and I189P showing trend towards slower responses. **d** Comparing relative response amplitude and decay of Rod opsin mutants shows that the two parameters are not strongly correlated. Data shown are mean ± standard error of mean of *n* = 9–11 replicates from 3 separate transfections. In **c**–**d,** a two-tailed one-sample Wilcoxon signed ranks test was used to compare each condition relative to Rod control (theoretical median = 1). *p < 0.05, **p < 0.01; ***p < 0.001, not significant where no asterisk is displayed. Grey asterisk = significant for uncorrected alpha (0.05), black asterisk = significant for Sidak corrected alpha (0.007 for *A* and *T*_*off*_)

We next explored increasing the rate of Schiff base hydrolysis as an approach to modifying *T*_*off*_. We used several previously described rod opsin mutants with faster meta-II decay [10, 25, 31, 38]. Using immunohistochemistry, we confirmed that all rod opsin mutants were expressed in HEK293T cells (Supplementary Fig. 5). Fluorescence intensity of anti-rod opsin 1D4 staining for rod opsin mutants was generally lower than for RodWT, except for A132S, which was comparable (Supplementary Fig. 3b). However, using live cell assays, we found that two rod opsin mutants, Y223F and A132L, were non-functional, being unable to cause a detectable change in BRET ratio upon light exposure (Fig. 5a). The remaining opsin mutants were functional, with most displaying response amplitudes attenuated to 24–48% of wildtype control (Fig. 5b). Y74F had comparable response amplitude to RodWT, while Y306F increased response amplitude from *A* = 0.47 ± 0.02 for RodWT to 0.52 ± 0.02 for Rod Y306F.

The impact of *T*_*off*_ of the rod opsin mutants was more complex (Fig. 5c). Of the 5 mutants with small amplitude responses, two demonstrated improved deactivation kinetics: E122Q and Y136F (*T*_*off*_ = 248.36 ± 41.6 and 379.55 ± 110.1 respectively). Deactivation of E122Q and Y136F was faster than RodWT (*T*_*off*_ = 504.19 s ± 115.9), with *T*_*off*_ 40% and 27% shorter than RodWT, respectively. Perhaps unexpectedly, the rod opsin mutant with largest response amplitude, Y306F, showed modestly faster deactivation, with T_off_ 70% of RodWT level (*T*_*off*_ = 256.73 ± 13.96). The remaining mutants had no significant effect on *T*_*off*_. Indeed, across the panel of rod opsin mutants, there was no correlation (Pearson’s *R* =  − 0.182, *p* = 0.1153) between response amplitude and decay (Fig. 5d), suggesting that targeting spontaneous opsin deactivation may represent a viable approach for optimising both temporal resolution and response amplitude.

We next tested whether it was possible to further improve temporal resolution by introducing E122Q, Y74F, Y136F, or Y306F mutations to the Rod6A-10 nm-Arr3A construct (Supplementary Fig. 6). We found that response parameters *A* and *T*_*off*_ were similar for Rod6A-10 nm-Arr3A with and without meta-II mutants, regardless of which mutation was used. Thus, our data do not reveal an advantage of co-applying both strategies.

### E122Q provides improved temporal resolution for visual restoration

**Fig. 6:**
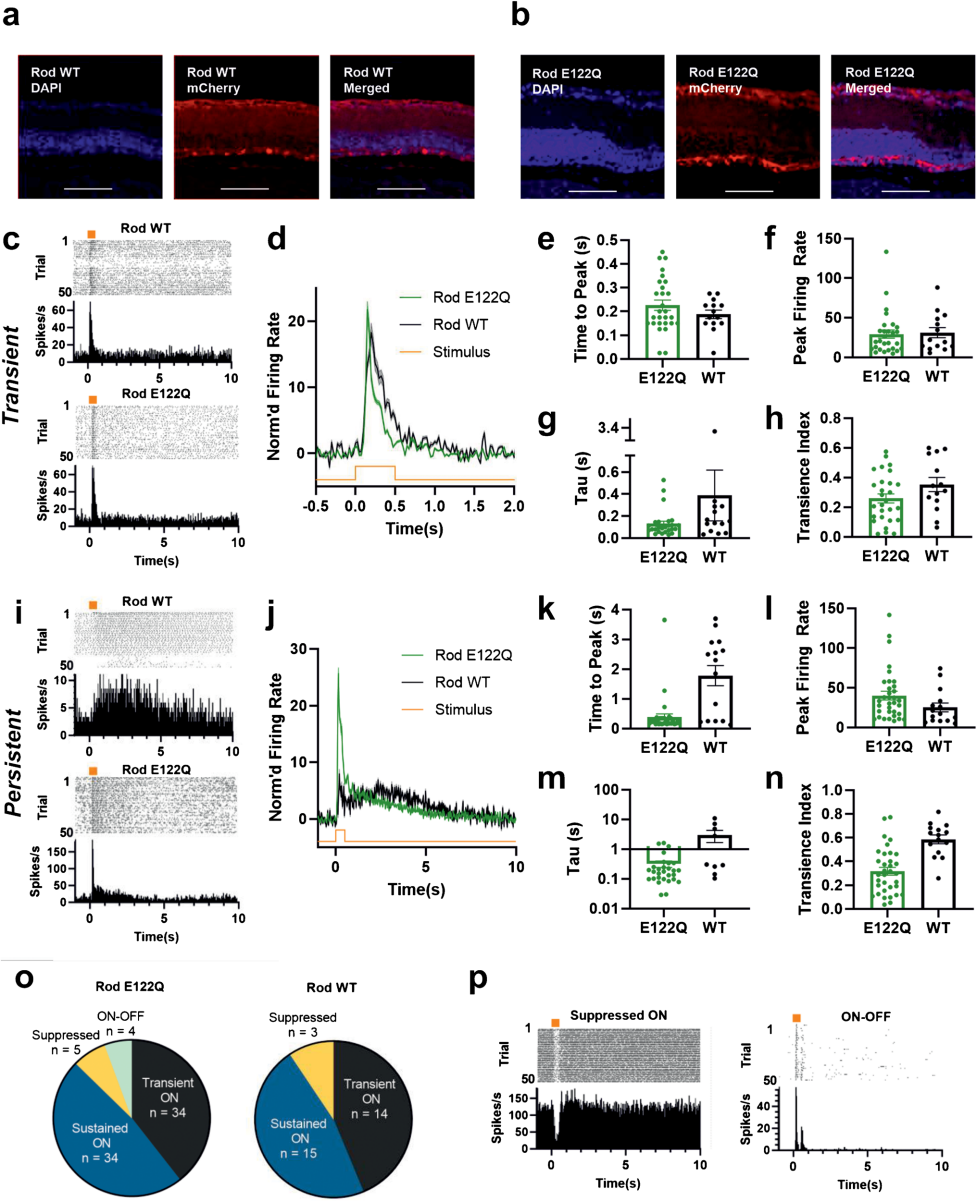
**a** Immunohistochemistry of retinal cross-sections from mice with intravitreal injection of AAV2 4YF- RodWT-T2A-mCherry or **b** AAV2-4YF Rod E122Q-T2A-mCherry (DAPI = blue, mCherry = red). Scale bar = 100 *µ*m. **c** Representative examples of units with transient responses during MEA recordings of retinal explants in response to 500 ms flash of light at 14.5 log effective Rod opsin photons/cm^2^/s. **d** Mean time-course of baseline-normalised firing rate for transient retinal units (*N* = 14 for RodWT and *N* = 28 for Rod E122Q unless stated otherwise). **e** Time to peak (s) for transient units.**)** Maximum firing rate (spikes/s) for transient units. **g** Rate of response decay calculated as tau for best-fit exponential decay curve (*N* = 26 for E122Q and *N* = 14 for WT). **h** Transience index for transient units. **i** Representative examples of units with persistent responses. **j** Average time course of baseline-normalised firing rate for persistent units (*N* = 15 for RodWT and *N* = 34 for Rod E122Q unless stated otherwise). **k** Time to peak (s) for persistent units. **l** Maximum firing rate (spikes/s) for transient units. **m** Rate of response decay calculated as tau for best-fit exponential decay curve (*N* = 29 for E122Q and *N* = 9 for WT). **n** Transience index for persistent units. **o** Distribution of different response types found in Rod E122Q (left) and RodWT (right) retinas. **p** Representative units from E122Q retina for suppressed ON (left) and ON–OFF (right) response types. Representative units shown in **c**, **i**, and **o** show perievent rasters (top) and firing rate histograms (bin size = 25 ms, bottom). Orange bars and lines show stimulus timing. Sample size (*N*) are for individual retinal units

The goal of controlling response lifetime is to alter performance in optogenetic applications. To determine whether the approaches outlined above were, in principle, suitable for this application, we next set out to determine whether one of the manipulations to rod opsin function could improve the temporal resolution of restored vision in advanced retinal degeneration. For this purpose, we turned to the E122Q mutation, which has several theoretical advantages for this application (see discussion) and performed well in the BRET assay. We used intravitreal injection of a recombinant viral vector (AAV2 4Y-F) to introduce a transgene comprising a ubiquitous promoter upstream of either a double-floxed inverse orientation (DIO) RodWT or Rod E122Q coding sequence into surviving inner retinal neurones in mice with advanced retinal degeneration (*Pde6b *^*rd1/rd1*^), expressing Cre-recombinase under the *Grm6* promoter. The result was expression of opsin in the outer nuclear layer, consistent with the reported ability of the *Grm6*^*cre*^ driver to target expression to ON bipolar cells (Fig. 6a–b). We, and others, have previously shown that expression of opsins (including rod opsin) in ON bipolar cells can restore visual responses at electrophysiological and behavioural level [9, 11, 23, 24, 35, 69]. To compare the visual response properties provided by RodWT and E122Q, we made extracellular electrophysiological recordings from the ganglion cell layer of retinal explants using a multi-electrode array.

We first identified light-responsive units that showed a statistically significant change in firing to a 500-ms flash (see Methods for details). Across 72 units from Rod E122Q (from 4 retinas from 4 mice) and 34 units from 6 RodWT-expressing retinas (from 3 mice) showing a significant response, we found a variety of distinct response profiles. The first response category was an increase in firing restricted to the 500 ms of stimulus presentation, defined hereafter as ‘transient’ responses (*N* = 28 for Rod E122Q, *N* = 14 for RodWT; Fig. 6c–d). Among this group, there was no statistically significant difference in either time to peak (median = 0.19 s for WT and E122Q, *U* = 167.5, *p* = 0.452, statistical comparisons use Mann–Whitney *U*-test unless stated otherwise), peak firing rate (median = 20.8 for WT and 20.2 spikes/s for E122Q, *U* = 176, *p* = 0.607), rate of response decay (median tau = 0.15 s for WT and 0.09 s for E122Q, *U* = 131, *p* = 0.153), or response persistence (median transience index = 0.33 for WT and 0.23 for E112Q, *U* = 139, *p* = 0.133) between wildtype and mutant rod opsin (Fig. 6e–h).

The second category of response was an increase in firing that persisted at least 500 ms after stimulus termination (Fig. 6i–j, N = 15 for RodWT, *N* = 38 for Rod E122Q). We termed such responses ‘persistent’. Units meeting the criterion of ‘persistent’ responses were found in both treatment groups; however, a closer inspection revealed qualitative differences in response within this class (Fig. 6k–n). Whereas persistent responses in E122Q peaked during or soon after stimulus presentation, in RodWT, they commonly built up more gradually (Fig. 6k, median time to peak = 2.3 s for WT and 0.25 s for E122Q, *U* = 114, *p* = 0.002). Response amplitude was also significantly higher in E122Q retinas (median = 17.2 for WT and 32.3 spikes/s, *U* = 163, *p* = 0.046, Fig. 6l). Rate of response decay was also significantly faster (median tau = 0.18 s for E122Q and 1.3 s for WT, *U* = 65, *p* = 0.004, Fig. 6m), and responses were significantly more transient (median transience index = 0.29 for E122Q and 0.62 for WT, *U* = 68, *p* < 0.001) in E122Q compared to WT retinas (Fig. 6n).

In addition to these common excitatory response types, we also found units with quite different characteristics (Fig. 6o–p). A small number of units in both treatment groups had suppressed firing during stimulus presentation (‘suppressed ON’), and in the E122Q retinas, we also found units with peaks at both start and end of the flash (‘on/off’).

**Fig. 7:**
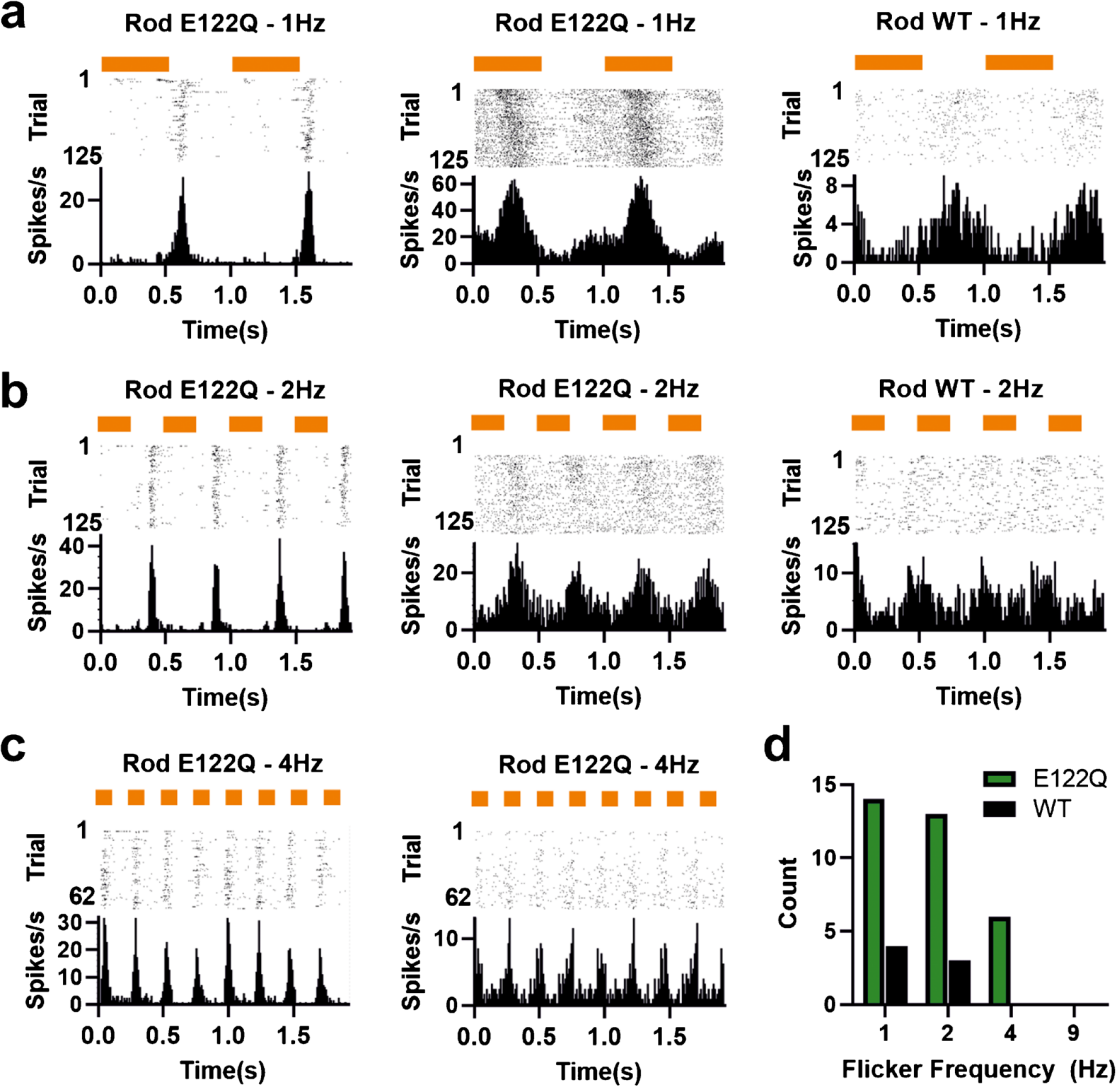
Representative units from MEA recordings from AAV2-4YF Rod E122Q (left and middle columns) and AAV2- 4YF RodWT (right column) injected retinas. Responses are to square wave (on/off) cycles at **a** 1 Hz, **b** 2 Hz, and **c** 4 Hz, at 14.5 log effective Rod opsin photons/cm^2^/s. **d** Number of units with significant oscillation matching stimulus frequency for 1, 2, 4, and 9-Hz flicker. Representative units shown in **a–c** show perievent rasters (first trial at top) and firing rate histograms (bin size = 10 ms). Orange bars show stimulus timing

Due primarily to the difference in properties of the ‘sustained’ group, there was an overall improvement in the temporal fidelity of the flash response in E122Q retinas. To determine whether this translated into an improvement in temporal resolution, we recorded responses to square-wave modulations in light intensity across a range of frequencies (1–9 Hz). A subset of light responsive units in both treatment groups showed a significant modulation in firing to the 1 Hz stimulus. Modulations in firing could be either quite sinusoidal in form or (especially in the E122Q group) more discontinuous (Fig. 7). The fraction of light-responsive units tracking the stimulus was similar between treatments at the lowest frequency (19% for RodWT vs 12% for E122Q, Fig. 7a), but this equivalence was lost at higher frequencies, with only 9% of RodWT light response units tracking 2 Hz and none at 4 Hz, while for the E122Q, these figures were 18% and 8% respectively (Fig. 7b–d). No unit from either group met our criteria for significant tracking of the 9 Hz stimulus.

## Discussion

Our experiments confirm that it is possible to engineer the temporal kinetics of animal opsin-driven signalling in live cells and that this can have advantages in optogenetic application. We find that this can be achieved by either enhancing arrestin-opsin interactions or the rate of Schiff base hydrolysis in the activated opsin. We found co-expression of factors designed to increase arrestin binding, and opsin point mutations both reduced the lifetime of rod opsin’s meta-II state and increased *T*_*off*_ in our BRET assay.

The BRET assay, validated for opsins here, reports the first step in opsin signalling (dissociation of G-protein heterotrimer) and thus provides a closer measure of the near instantaneous changes in opsin state than alternatives based upon second messenger reporters. However, it still provides an indirect measure of opsin activity, and the changes in *T*_*off*_ induced by our manipulations should be viewed in this context. *T*_*off*_ is likely influenced by delays in the accumulation and decline of free G_by_ and in the association/dissociation of nanoluciferase-tagged GRK3 fragment with G_by_, meaning that there need not be a simple linear relationship between *T*_*off*_ and the rate of decay of signalling active opsin. This precludes quantitative extrapolations from the data collected here to the lifetime of signalling opsin. Nevertheless, the differences we observe confirm that the assay can reveal changes in the lifetime of activated opsin (at least over the range explored here) with respect to a suitable positive control. In this regard, almost all the manipulations attempted here decreased *T*_off_, confirming the utility of our current understanding of the steps in rod opsin signal termination.

One reasonable concern is that reductions in *T*_*off*_ observed here may reflect a fundamental deterioration in opsin signalling efficiency rather than a specific change in activation lifetime. Particularly in the case of engineered opsins, this could arise from reductions in protein stability or subcellular mislocalisation. Our histological analysis confirms that all our opsin constructs are expressed at detectable levels, but lacks the resolution to determine the fraction of protein that is successfully localised to the plasma membrane. A more comprehensive analysis of the subcellular localisation of these opsin proteins is thus an important goal for future work. Turning to the broader concern that our manipulations have simply reduced signalling efficiency, there is a general tendency for our manipulations to both reduce response amplitude (*A*) and increase *T*_*off*_. This could indeed be partially attributable to non-specific effects of our manipulations on opsin activity (although the nature of the G_by_ accumulation assay is itself an obvious potential origin for this correlation). Nevertheless, across our dataset, there were examples of manipulations that both increased response amplitude and improved temporal resolution (Arr3A co-expression and Y306F point mutation) or vice versa (co-expression with GRK1). This confirms that our assay can detect changes in both *A* and *T*_*off*_ and that these can be mechanistically distinct effects.

The biggest improvements in *T*_*off*_ were achievable by targeting arrestin binding. Like all GPCRs, opsins are deactivated in a two-step process in which phosphorylation facilitates binding to arrestin, which inhibits signalling. We identified an important challenge in applying this strategy to opsins during heterologous expression in the form of a strong suppressive effect on peak light response amplitude when expressing the kinase required for phosphorylation (GRK1). This is consistent with the known suppressive effects of GRK1 on rod opsin activity, revealed as larger amplitude responses in mice with genetically ablated *GRK1* [14] and a biphasic response (large initial response, followed by rapid decrease to more sustained lower level after phosphorylation) in *Arr1*^*−/−*^ rod photoreceptors [31]. This dramatic reduction in response amplitude because of receptor phosphorylation is not found in all GPCRs. For example, beta-2 adrenergic receptor [63] and angiotensin II type 1A receptor [62] both show faster responses without substantial loss of response amplitude in the presence of beta-arrestin and GRK2. This likely reflects the action of the specific receptor kinase and arrestin binding to the GPCR, which appears to differ for visual and non-visual receptor desensitisation. We were able to overcome this problem by applying arrestin mutants with enhanced affinity for unphosphorylated active receptor (especially Arr3A). These effectively reduced response lifetime in the absence of GRK1, avoiding the suppressive effects of GRK1 expression on response amplitude. A further theoretical advantage of Arr3A is that it works on the Rod6A mutant. As Rod6A is less subject to quenching by native arrestins, the combination of Rod6A and Arr3A could make opsin signalling lifetime less dependent on the characteristics of the cell in which it is expressed and more reproducible across applications.

We found that tethering Arr3A to the opsin protein allowed faster response decay. Indeed, tethering Arr3A to Rod6A with a 10-nm linker provided the best combination of high *A* and fast *T*_*off*_ of any or our manipulations. In addition to its advantages in terms of *T*_*off*_ reduction, tethering may also mitigate potential problems associated with employing the phosphorylation-independent arrestin mutants. First, tethering to opsin is expected to reduce the potential of these introduced arrestins to interfere with native G-protein-coupled receptors and their separate signalling cascades. Second, overexpression of Arr3A mutant in mouse rods caused photoreceptor degeneration indicating that it may be cytotoxic [59]. The deleterious effects of this mutant in photoreceptors are believed to be due to reduced self-association of Arr3A, leading to high concentration of monomer units which interact with signalling pathways that can cause apoptosis [55]. To what extent the cytotoxicity of these mutants is limited to rod photoreceptors, which have unique morphology and protein expression (possessing 1000–10,000 more signalling proteins) [50], is unclear. Tethering the arrestin to rod opsin may limit the off-target effects of Arr3A and ameliorate potential cytotoxicity.

One potential limitation of the arrestin strategy for optogenetic applications is that it places a greater burden on the packaging size of viral vectors. Application of Arr3A precludes the need to co-express a rhodopsin kinase, but it still requires the virus to carry two coding sequences rather than just the opsin. In this regard, strategies to increase the rate of meta-II decay by altering the opsin sequence have an advantage. In native rod opsin, decay of the signalling meta-II state by Schiff base hydrolysis is relatively slow, on the order of minutes [6, 57, 65]. We tested a range of opsin mutants shown by spectroscopic methods [25, 31, 38] to accelerate meta-II decay to a variety of extents, from 0.2–0.5-fold (Y74F, Y136F,and Y306F) to 3-7 fold (A132L, E122Q, and Y223F). Two mutants with very fast meta-II decay (A132L and Y223F) failed to show a detectable light response in the BRET assay, perhaps because their activation lifetime is too short for efficient G-protein activation in live cultured cells. Of the remaining, E122Q had the largest effect on *T*_*off*_, consistent with the published spectroscopic data [25, 31, 38].

While both approaches could shorten the secondary messenger profile (Supplementary Fig. 3c–d), we chose the E122Q mutant as our prototype for testing the translatability of the findings from the BRET assay to a genuine optogenetic application. We did so because it had the fastest *T*_*off*_ of any of the mutants, and in preference to the arrestin strategies because of its small size for viral packaging. Moreover, there is a wealth of data about the functional consequence of E122Q for opsin structure and photochemistry, and it has been shown to be non-cytotoxic, at least in photoreceptors [27]. Several aspects of the visual response driven by E122Q in *rd1* retina are consistent with an increase in temporal fidelity over that produced by RodWT. Firstly, there is an overall reduction in longevity of a response to a 500-ms flash. In RodWT-treated retinas, a substantial proportion of visually responsive units showed gradual and long-lasting increases in firing to the flash, not seen in the E122Q units, as demonstrated by the faster rate of response decay and lower transience index values compared to RodWT. Time-delimited ‘transient’ responses were found in RodWT-treated retinas, but as we targeted expression to ON bipolar cells, this transience could plausibly arise by temporal bandpass filtering at the bipolar cell-ganglion cell synapse or at other points in inner retinal circuitry. Another indication of improved temporal resolution with E122Q was the higher fraction of visually responsive units which tracked flickering stimuli at frequencies greater than 1 Hz. Previous work has proposed improved temporal resolution using cone opsins for visual restoration [9] or *in vivo* control of brain neurons [43]. Our work implies that at least a portion of that effect is attributable to reduced Schiff-based stability (a property shared by cone opsins and E122Q).

Relatively poor temporal fidelity is one of the major disadvantages of animal opsins compared to microbial opsins as optogenetic actuators. Our work here confirms that this problem is at least partially addressable by exploiting well-established mechanisms of opsin deactivation. The strategies used here should be applicable not only to mammalian rod opsin, but also to other animal opsins and to opsin/GPCR chimeras [1, 30, 40, 47]. They represent a complementary approach to the use of photocyclic [54] or bistable, switchable, opsins in which different wavelengths of light may be used to switch opsins on and off [18, 21, 32, 51].

## Methods

### Expression vector construction

pDNR-DUAL human rhodopsin kinase (NM_002929) was obtained from DNASU plasmid repository, where it was deposited by the Harvard Institute of Proteomics. pENTR223.1 human rod arrestin (NM_000541) was also obtained from DNASU plasmid repository, where it was deposited by the ORFeome collaboration. pcDNA3 human rod opsin (NM_000539.3), pcDNA3 Glo22F, and pcDNA3 GsO plasmids were as described previously [2, 5]. Human GalphaO_A_ (AH002708) with pertussis toxin resistant *Cys352Ser* mutation was purchased from the cDNAResource Center (www.cDNA.org). BRET G-protein activation assay constructs—pcDNA3 splitVenus-Gbeta1 (sVβ1), pcDNA3 splitVenus-Ggamma2 (sVγ2), and pcDNA3 mGRK3-nLuc were as described previously [44, 45]. Where necessary, ORFs were cloned into pcDNA3 vector using Gibson assembly.

Phosphorylation-independent mouse arrestin1 mutants were adapted from [64] for human arrestin1. Arrestin 3A mutant was created by introducing the following mutations—L377A, V378A, and F379A—into pcDNA3 Arrestin using Quikchange Lightning site-directed mutagenesis kit (Agilent) with the following primers: Arr1 3A Fwd 5′–GTTATCAGGATGCAAATgcAgcTgcTGAGGAGTTTGCTCGCC and Arr1 3A Rev 5′-GGCGAGCAAACTCCTCAgcAgcTgcATTTGCATCCTGATAAC. The ArrestinKEQ3A mutant was created by introducing additional mutations—K261Q, E350H, and Q332K—to pcDNA3 Arrestin 3A using Quikchange multisite-directed mutagenesis kit (Agilent) using the following primers: K261Q Fwd 5′–CGAGTGATTATTACGTCcAGCCCGTGGCTATGGAG, K261Q Rev 5′–CTCCATAGCCACGGGCTgGACGTAATAATCA CTCG, Q332K Fwd 5′–GAATCCTGGTGTCTTACaAGATCAAGGT.

GAAGCTCAC, Q332K Rev 5′–GTGAGCTTCACCTTGATCTtGTAAGACACCAGGATTCC, E350H Fwd 5′–GAGAGCTCACCTCCAGTcAcGTCGCCACTGAGGTCC, and E350H Rev 5′–GGACCTCAGTGGCGACgTgACTGGAGGTGAGCTCTC.

For construction of rhodopsin6A expression vector, a 246 bp DNA fragment was synthesised by Thermo Fisher, which corresponded to the final 196 bp of the human rhodopsin ORF and a 50 bp overlap with pcDNA3 backbone from *NotI* site. This fragment possessed 6 mutations (S333A, T336A, S338A, T340A, T342A, S343A) designed to remove phosphorylation sites from the rhodopsin C-terminus. This fragment was cloned into pcDNA3 rhodopsin vector linearized with *AfeI* and *NotI* using Gibson Assembly.

Arrestin3A or Arrestin KEQ3A were cloned in-frame after rhodopsin6A by amplifying arrestin ORF using the following primers: Arr Fwd 5′-caggtggccccggcTaCGCGtGCAGCCAGCGGGAAG ACCAGC and Arr Rev 5′–caggaattcgatatcaagcACCGGTTTACTCATCAACGTCATTCTTGTC TCTC. The forward primers introduced a 6 bp *MluI* restriction site between Rhodopsin6A and arrestin mutant coding sequences. To construct bicistronic vectors or fusion constructs between Rhod6A and Arr3A/ArrKEQ3A, DNA sequences corresponding to desired linker or P2A sequence were synthesised by Thermo Fisher and cloned into pcDNA3 Rhod6A-MluI-Arr3A or pcDNA3 Rhod6A-MluI-ArrKEQ3A linearised by *MluI* digest and treated with recombinant shrimp alkaline phosphatase (NEB) using Gibson assembly.

The following linkers were used in fusion constructs: Flexible linkers composed of (GGGGS)_n_ units where *n* = 1–3 (referred to as F1–F3, respectively), rigid linkers composed of A(EAAAK)_n_A units where *n* = 1–3 (referred to as R1–R3, respectively), and semi-flexible linkers composed of GGGGSA(EAAAK)_n_AGGGGS where *n* = 1–5 (referred to as SF1–SF5 respectively). We also tested fusion constructs using the 10 nm and 20 nm E/RK α-helix linkers described previously [58]. For the E/RK α-helix linkers, an additional (GSG)_4_ motif was included at the 5′ and 3′ end of E/RK linker to ensure rotational freedom.

Presence of mutations and cloned ORFs was confirmed by Sanger sequencing.

### Cell culture and transfections

HEK293T cells (ATCC Cat# CRL-3216, RRID:CVCL_0063) were incubated at 37 °C (5% CO_2_) in culture media (Dulbecco’s modified Eagle’s medium with 4500 mg/L glucose, L-glutamine, sodium pyruvate, and sodium bicarbonate from Sigma) and 10% foetal bovine serum (FBS).

For transfections, cell were seeded into 12-well plates at a density of 250 000 cells/well in culture medium. After 48 h, cells were transiently transfected using Lipofectamine 2000 (Thermo Fisher) according to manufacturer’s instructions. For all transfections, total amount of DNA was normalised between conditions using empty vector.

For BRET G-protein activation assays described in Figs. 1, 2, S1, and S2, each well of 12-well plate was transiently transfected as follows: 100 ng sVβ1, 100 ng sVγ2, 100 ng mGRK3-nLuc, 200 ng Gαo, 500 ng opsin, and where appropriate 500 ng arrestin (or arrestin mutant) and/or 500 ng rhodopsin kinase. The ratio and amount of BRET assay components were as described in [44, 45].

After completing these initial experiments, we optimised the amount and ratio of each of the BRET assay components to improve reliability of assay. We did this by measuring BRET ratio of different levels of mGRK3-nLuc in isolation and then for optimised GRK3-nLuc in combination with different amounts of sVβ1 and sVγ2 and finally different ratios of Gαo to optimised amounts of sVβ1, sVγ2, and mGRK3-nLuc. For these optimised transfection conditions, used to collect data shown in Figs. 4, 5, S3, and S5, each well of 12-well plate was transfected as follows: 100 ng sVβ1, 100 ng sVγ2, 25 ng mGRK3-nLuc, 50 ng Gαo, and 500 ng opsin or opsin-arrestin fusion.

For all BRET experiments, once transfected, subsequent steps were conducted under dim red light. After addition of transfection, reagent and DNA cells were incubated for 4–6 h at 37 °C and then resuspended in 1 ml of culture media containing 10 μM 9-*cis* retinal (Sigma-Aldrich). 100 μl of resuspended cells were added to each well of a white-walled clear-bottomed 96-well plate (Greiner Bio-One) and left overnight before performing BRET G-protein activation assay.

For Glosensor cAMP assay, each well of 12-well plate was transfected as follows: 500 ng opsin (or opsin-arrestin fusion), 500 ng Glo-22F, 5 ng GsO, and where appropriate 500 ng arrestin (or arrestin mutant) and/or 500 ng rhodopsin kinase. After addition of transfection, reagent and DNA cells were incubated for 4–6 h at 37 °C and then resuspended in 1 ml of culture media containing 10 μM 9-*cis* retinal and 125 ng/ml pertussis toxin. 100 μl of resuspended cells was added to each well of a white-walled white-bottomed 96-well plate (Greiner Bio-One) and left overnight before performing cAMP assay.

### BRET G-protein activation assay

Approximately 1–2 h before beginning BRET G-protein activation assay, culture media was removed from cells and replaced with 50 μl imaging media (L-15 media without Phenol Red containing L-glutamine (Gibco), 1% FBS, penicillin (100 U/ml), and streptomycin (100 μg/ml) with 10 μM 9-*cis* retinal. Cells were then left to incubate at room temperature in dark for at least 1 h.

Under dim red light, NanoGlo Live Cell substrate (Furimazine derivative, Promega) was diluted 1:40 in PBS. Then, 12.5 ul of dilute NanoGlo substrate solution was added to each well of 96-well plate (to provide final dilution of 1:200 of NanoGlo substrate), for up to 6 wells at a time, and incubated for 5 min before commencing assay to allow luminescence to peak. We found recordings conducted more than 20 min after cells were initially loaded with substrate tended to be noisier as overall BRET signal decreased.

BRET measurements were conducted using a FluoStar Optima microplate reader (BMG Labtech). As this plate reader has a single photomultiplier tube, light emitted by fluorescent Venus and bioluminescent Nanoluc were measured sequentially using 535 nm (30 nm FWHM with gain set to 4095) and 470 nm (30 nm FWHM with gain set to 3600) emission filters. A 0.68-s recording interval was used for each filter, with a total cycle time of 2 s.

To avoid delays associated removing plate from reader for light exposure, we adapted the plate reader bottom optic to allow us to deliver light to individual wells inside the plate reader. A custom 3D-printed coupler was used to connect the bottom optic with the liquid light guide of a Lumencor SpectraX light engine. Combined with clear-bottomed 96-well plates, this allowed us to provide a light stimulus below cells. To avoid bleaching the PMT during light stimulus, a motorized shutter was built to protect the PMT by blocking top optic light path while light stimulus was on. The activity of this shutter was synced to the light source using an Arduino microcontroller. To avoid neighbouring wells being exposed to light, each recorded well was surrounded by empty wells, and the order of wells measured was counterbalanced.

During BRET plate reader recordings, using Optima script mode, a 16.5-s pause followed by a 1-s 485-nm light pulse (16.5 log photons) was first triggered by an executable file. A well-mode protocol for the individual well to be recorded was then immediately started. This protocol consisted of 2 kinetic windows; the first consisted of 5 cycles of baseline measurement (total duration 10 s), and the second protocol began after a short 3–4-s delay (during which cells were exposed to the delayed light pulse), before continuing for up to 45 cycles (total duration 90 s). The pause between the two kinetic windows was varied according to the well position being recorded in order to account for different delays in time taken to travel from plate reader ‘home position’ and ensure that the recording was resumed immediately after light flash. This process was then repeated for until all wells loaded with substrate had been measured. In each recording session, between 3 and 4 repeats were conducted for all conditions. At least 3 recording sessions (each a separate transfections) were performed for each experiment.

### Glosensor Gso cAMP assay

Glosensor Gso assay was performed as described previously [5]. Briefly, 1–2 h before beginning assay, cells were incubated at room temperature in 75 ul imaging media with 2 mM beetle luciferin potassium salt (Promega) reconstituted in 10 mM HEPES pH 6.9. Using the FluoStar Optima microplate reader, raw luminescence was recorded using 3-mm lens (Gain set to 3600) for 1 s, every 60 s. Baseline luminescence was recorded for 5 cycles, and then recording was paused, and plate ejected. Each well was then stimulated with 470-nm light flash using a custom-built LED array. Each well was exposed to one of eight different intensities over a 5-log range (from 4 × 10^11^ to 10^15^ photons). One well from each condition was left unexposed as a dark control. Normalised Glosensor response shown in Fig. 1b was calculated by dividing each raw luminescence data point by the last baseline luminescence value before stimulus onset.

### Data analysis

For irradiance response curves (IRCs) from cAMP assay, raw luminescence data was normalised by dividing each data point by the last baseline luminescence value before stimulus. The response amplitude was calculated as the maximum fold-increase from baseline observed after stimulus exposure. The response amplitude for the 8 different intensities of 470-nm light tested was fit with a sigmoid IRC using non-linear regression. The IRC was defined by following three parameter equation: *y* = *a* + *b*(1 + 10^(*c* – *x*)^), where *a* is baseline, *b* is response amplitude, and *c* is logEC50. LogEC50 was used to compare sensitivity between different opsin constructs. We tested *n* = 3–4 biological replicates at each intensity for each construct. An IRC was then fit to each biological replicate, and mean LogEC50 for each condition was compared between groups.

For BRET G-protein activation assay, BRET signal was determined by calculating ratio of light emitted by Venus-Gβ_1_γ_2_ at 535 nm with light emitted by mGRK3-nLuc at 470 nm. The BRET signal was then normalised to baseline by dividing each time point by the last baseline value before stimulus to give ΔBRET ratio. The kinetics of ΔBRET ratio time course post-stimulus were then fit to the following 3-parameter model using nonlinear regression:

$${y=e}^{\left({-T}_{off}/X\right)}{\blacksquare} \left({1-e}^{\left({-T}_{on}/X\right)}\right)\blacksquare A$$

where *T*_*off*_ = rate of decay of exponential decay curve, *T*_*on*_ = rate of increase of exponential association curve and* A* = scaling factor of two exponential curves, *x* = time (seconds), and *y* = baseline normalised BRET signal. The model was fit to data using nonlinear regression. The following constraints were used: A > 0, *T*_*on*_ > 0.1, *T*_*off*_ > 5. A goodness of fit threshold of *R*^2^ > 0.2 was used. Curve fits produced by GraphPad Prism that were ambiguous or did not converge were excluded from further analysis and are not included in time courses displayed in figures (except where all data is displayed). Scaling factor (A) was used as a measure of response amplitude, while *T*_*off*_ (s) was used as a measure of response decay. The 3-parameter model was used for all conditions except the RodWT + GRK1 (Fig. 1d), where 4 out of 9 replicates failed to produce adequate curve fits. Instead, the RodWT + GRK1 data was modelled with a one-phase exponential association curve (2 parameters, *Ton* and *A*), which was used to calculate amplitude value shown in Fig. 1g.

We found systematic variation in overall ΔBRET ratio of different recordings, likely driven by differences in transfection efficiency, as well as density, health, and total number of cells. To account for this, we looked at fold change in *A* and *T*_*off*_ of each condition relative to RodWT positive control conducted during the same repeat. Response amplitude and decay were then analysed using Wilcoxon signed-rank test, comparing each group to theoretical median = 1.

### Immunocytochemistry

HEK293T cells were seeded into 12-well plates at a density of 250 000 cells/well in culture medium. After 48 h, cells were transiently transfected using lipofectamine 2000 (Thermo Fisher) according to the manufacturer’s instructions with 500 ng opsin or opsin-arrestin fusion. Cells were incubated at 37 °C for 4–6 h and then, under dim red light, resuspended in 2 ml of culture media containing 10 μM 9-*cis* retinal (Sigma-Aldrich). The entire volume of resuspended cells was then added to a well of a 6-well plate containing 3 × poly-D-lysine coated glass coverslips. Cells were incubated for further 24 h and then washed once with PBS, before being fixed using 4% paraformaldehyde in PBS. Cells were then washed three times in PBS and stored in PBS at 4 °C until being stained.

For staining, one coverslip per condition was removed and placed in each well of 12-well plate. Cells were permeabilised in 0.2% Triton-X in PBS for 5 min and then blocked in PBS + 0.05% Tween-20 with 5% serum for 20–30 min. Cells were incubated in primary antibodies diluted in PBS + 0.05% Tween-20 + 1% serum for 1 h at room temperature and then washed three times in PBS. Cells were then incubated in secondary antibody diluted in PBS + 0.05% Tween-20 + 1% serum for 30 min at room temperature in dark. Cells were washed in PBS 3 more times, and then each coverslip was mounted onto slides using Prolong Gold anti-fade media with DAPI and allowed to dry at room temperature for at least 24 h.

The following primary and secondary antibodies were used: Mouse monoclonal anti-4D2 N-terminal rod opsin antibody 4D2 (1:500, Abcam Cat# ab98887, RRID:AB_10696805) with Donkey anti-mouse far red 594 secondary (1:500, Molecular Probes Cat# A-21203, RRID:AB_141633) with donkey serum and Mouse monoclonal anti-1D4 C-terminal rod opsin antibody (1:500, Abcam Cat# ab5417, RRID:AB_304874) with Goat anti-mouse red 555 secondary (1:500, Molecular Probes Cat# A-21127, RRID:AB_141596) with Goat Serum.

Images were acquired using an Axio Imager.D2 Upright microscope (Zeiss) using a 40 × plan neofluar air objective, using excitation at 350 nm, 545 nm, and 580 nm and emission at 460 nm, 605 nm, and 650 nm for DAPI, red, and far red fluorescence, respectively. Images were collected using Micromanager v1.4.23, with a Coolsnap HQ2 camera (Photometrics) and CoolLED pE-300 White light source. Images were analysed using ImageJ (RRID:SCR_003070) [56]. Global adjustments to brightness and/or contrast were applied equally to all images.

### Intravitreal injections

All experiments were conducted in accordance with the UK Animals (Scientific Procedures) Act (1986). *Grm6Cre* [46] transgenic mice on a mixed C3H × C57Bl/6 background were used for retinal MEA experiments. These mice express Cre recombinase in ON bipolar cells. They also possess the *Pde6b*^*rd1*^ mutation [12, 49], which causes progressive retinal degeneration, with vision loss complete once animals are over 80 days old. All mice were kept under a 12:12 light–dark cycle with food and water provided ad libitum. *Grm6*^*Cre/*+ ^*rd1* mice received bilateral intravitreal injections of RodWT or Rod E122Q virus (AAV2 4YF–DIO-CMV-RodWT-T2A-mCherry or AAV2 4YF–DIO-CMV-RodE122Q-T2A-mCherry). The virus was packaged using the 4Y-F AAV2/2 capsid [48] to achieve efficient viral transduction of retinal cells, in particular bipolar cells. Rod opsin was linked to a mCherry fluorescent reporter using a T2A sequence to ensure 1:1 co-expression of the two proteins. The opsin-T2A-mCherry open reading frame was double-floxed by LoxP and Lox2272 sites and inverted. In the presence of Cre, expression is driven by the constitutive CMV (cytomegalovirus) promotor. A woodchuck hepatitis virus post-transcriptional regulatory element (WPRE) and SV40 late polyA sequence were also included between ITRs to improve transgene expression. Virus was obtained from VectorBuilder.

### Multi-electrode array recordings from retinal explants

Recordings were conducted on 6 RodWT and 4 Rod E122Q injected *Grm6*^*Cre*^* rd1* on a 256 electrode array (multichannel systems). Retinas were dissected and supplemented with AMES media and 4 µM 9-Cis-retinal (Sigma) at a flow rate of 2.5 mL/min. A high pass filter of 200 Hz was utilised. The full field light stimuli were provided by a customised light source at 0.5-s flashes with a 10-s inter stimulus interval for the single flash dataset; flicker stimuli were symmetrical square wave (on/off) cycles at 1.01, 2.04, 4.16, and 9.09 Hz flashes all at an intensity of Log 14.5 effective Rod opsin photons/cm^2^/s. Principal component based spike sorting was used to extract the activity of single units from multi-unit recordings and was undertaken using Plexon Offline Sorter.

### Flash response analysis

For analysis of responses to 500-ms flash, we sampled firing in 25-ms bins. We identified light responsive units by correlating different trials and calculating the mean correlation coefficient between trials. We then randomly shuffled bins within each trial and then correlated each trial of shuffled data and generated a mean correlation value. This process was repeated 1000 times to produce a null distribution used for comparison with the original unshuffled mean correlation coefficient.

For analysis of response characteristics, data was first smoothed using Matlab *smoothdata* function (using ‘Gaussian’ window with width set to 5 bins). To classify units into different response categories, we examined where mean firing rate (spike/s) during a defined response window fell outside mean ± 3 standard deviations of baseline firing rate (1-s preceding flash onset). We used 3 different response windows: window 1 during flash (0–500 ms), window 2 immediately following flash (500 ms–1 s), and window 3 later after flash (1–2 s). Any units with spike firing in < 10% of bins were excluded from further analysis. We then sorted light responsive units into different categories as follows:

Transient excitation responses had significant excitation to window 1, significant excitation or no response to window 2, and no significant response to window 3. Persistent responses have no significant response or significant excitation to windows 1 and 2, with significant excitation to window 3. Transient inhibitory responses have significant inhibition in any of the 3 windows. We observed some units with biphasic responses (transient on and off responses) were incorrectly included in the sustained excitation category above. To separate these, we applied a further test comparing activity during 500 ms after flash offset with mean ± 2 standard deviations of firing rate during last 200 ms of flash. Transient excitation units with significant responses to this additional test were categorised as biphasic.

For calculation of rate of response decay, exponential decay curves were fit to data in either 2 s or 5 s after peak firing rate for transient and sustained responses respectively. Curve fits with *R*^2^ < 0.3 were excluded. The transience index was calculated using method described in Farrow and Masland [22]. Data was sampled in 100-ms bins and then normalised to maximum firing rate. The area under the curve was then calculated for 8 s after light onset and divided by total number of bins to produce measure of how similar responses are to peak firing rate across sampled response window. The resulting value, the transience index, ranges from 0 (highly transient) to 1 (highly sustained).

For flicker data, we generated a mean PSTH for 1.92 s of flicker data (2 cycles of 1.01 Hz, 4 cycles of 2.04 Hz, 8 cycles of 4.16 Hz, and 18 cycles of 9.09 Hz data) with 10-ms bin size. Light responsive units were identified as described above. We then generated a periodogram and checked whether peaks were detected at the stimulus of the frequency. If any peaks were detected, we checked whether these were significant by generating a Fischer’s G statistic (ratio of peak power to sum of all power values) and calculating a *p* value, using a method described in [67]. We used a Bonferroni correction for multiple comparisons to determine significance threshold (0.05/number of spiking channels). Units with significant oscillation at stimulus flicker frequency were classified as responsive.

### Immunohistochemistry of retinal sections

Eyes were collected and fixed in 4% paraformaldehyde in PBS overnight and subsequently cryopreserved in 30% sucrose overnight. Eyes were embedded in OCT and frozen on dry ice before being sectioned at 20 µM thickness. Sections were blocked in 5% Donkey serum in PBS with 0.1% triton-X before application of Rabbit polyclonal anti-mCherry antibody (Kerafast) to the slides at 1:400 in blocking solution at 4 °C overnight. After washing, Donkey anti-rabbit 546 was applied to the slides for 2 h at room temperature. Slides were mounted with ProLong Gold Antifade with DAPI (Thermo Fisher) and allowed to dry overnight. Images were acquired using a Leica DM2500 microscope with a Leica DFC365 FX camera. Samples were illuminated using a CoolLED-pE300-W light source filtered with Chroma ET A4 (for DAPI) and Y3 (for mCherry) filter sets. Global adjustments to brightness and contrast were applied equally to all images.

### Supplementary Information

Below is the link to the electronic supplementary material.

Supplementary file1 (PDF 1.70 MB)

## Data Availability

Raw data and materials will be made available upon reasonable request to corresponding authors, Jessica Rodgers (jessica.rodgers@manchester.ac.uk) and Robert Lucas (robert.lucas@manchester.ac.uk).
