# Supplementary material for: Modulating signalling lifetime to optimise a prototypical animal opsin for optogenetic applications
Source: Pflugers Arch. 2023 Dec 1;475(12):1387–407. doi: 10.1007/s00424-023-02879-9 (PMC10730688; doi:10.1007/s00424-023-02879-9)
Supplement: Supplementary file 1 — Supplementary file1 (PDF 1.70 MB) [file 424_2023_2879_MOESM1_ESM.pdf]

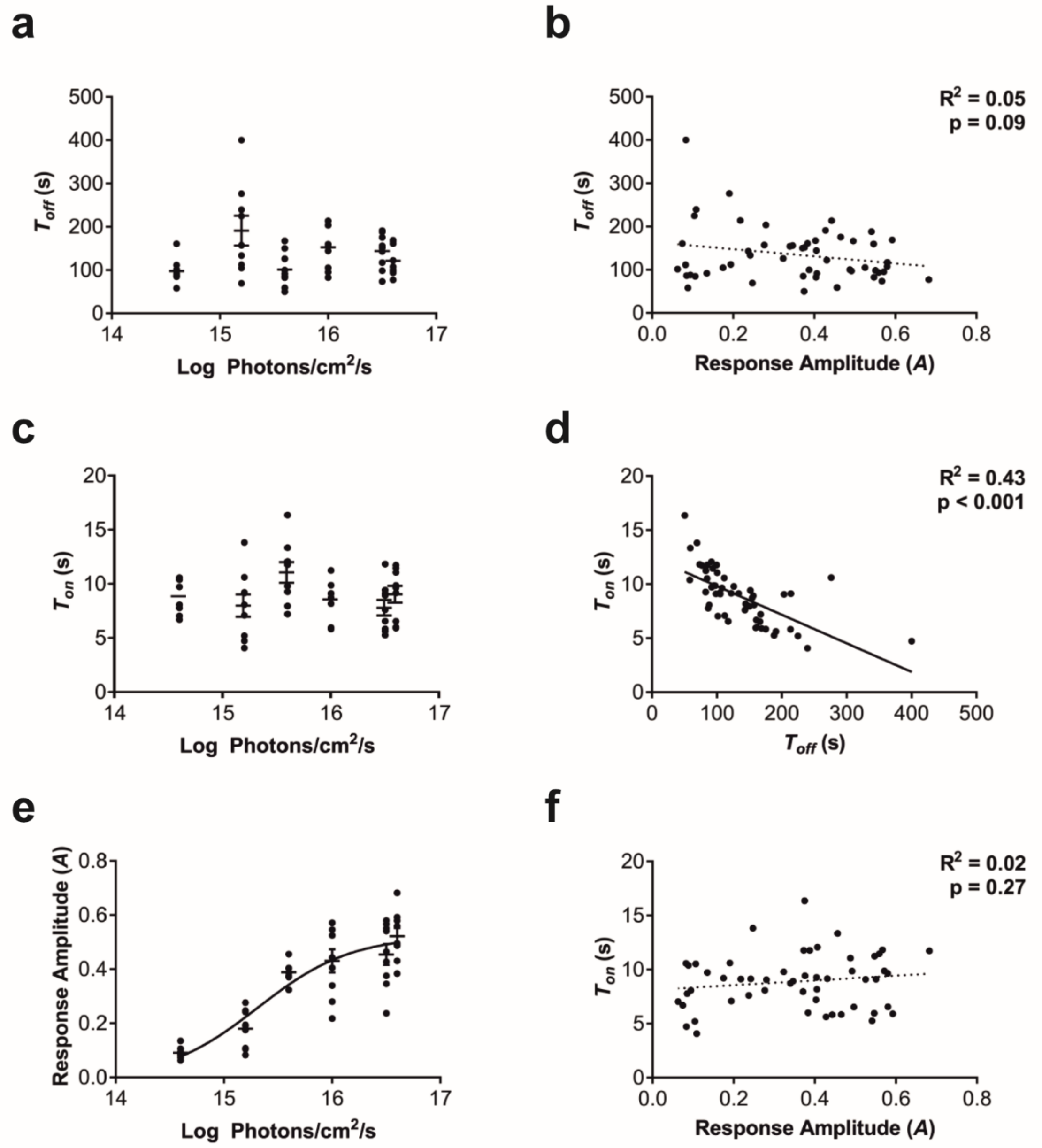

Supplementary Figure 1.

Rod opsin-driven G protein activation to 1s flashes of varying intensities (14.3 – 16.6 log photons) of 485nm light were measured using BRET assay and fit with simple 3 parameter model (*A*, T_{on} and T_{off}) using non-linear regression. The same opsin activated to different levels should have similar rates of response onset and response decay, **a)** The response decay rate (measured as T_{off}) is consistent across responses to different intensity flashes (ie: different levels of G protein activity).**b)** Response decay does not significantly correlate with response amplitude (Pearson's product-moment correlation, r = -0.23, R^2 = 0.05, p = 0.097) **c)** The response onset (measured as T_{on}) is also consistent across intensities but **d)** does significantly correlate with response amplitude (Pearson's product-moment correlation, r = -0.66, R^2 = 0.43, p < 0.001) **e)** Response amplitude is irradiance-dependent, consistent with increasing levels of G protein activity at higher intensities. These data can be fit with an irradiance response curve (IRC) with log EC50 = 15.31 log photons, R^2 = 0.74. **f)** Two parameters, T_{on} and T_{off}, are significantly negatively correlated with longer lifetime responses showing shorter response onset (Pearson's product-moment correlation, r = -0.66, R^2 = 0.43, p < 0.0001). For **a, c, e)** a model comparison (F-test) was performed to determine if data were better fit by a horizontal line (null hypothesis) or a sigmoid irradiance response curve (IRC, alternative hypothesis) using non-linear regression. For data where null hypothesis was rejected, such as response amplitude vs intensity, (F(2,50) =7086, p < 0.0001, the best fit IRC is displayed. For response decay and response onset vs intensity, null hypothesis was not rejected (p > 0.05). Error bars show standard error of the mean. For **b, d, f)** the best-fit linear trendline is displayed (solid line for significant correlations, dashed line for non-significant correlations). Data are from 3 independent experiments (3 technical replicates each).

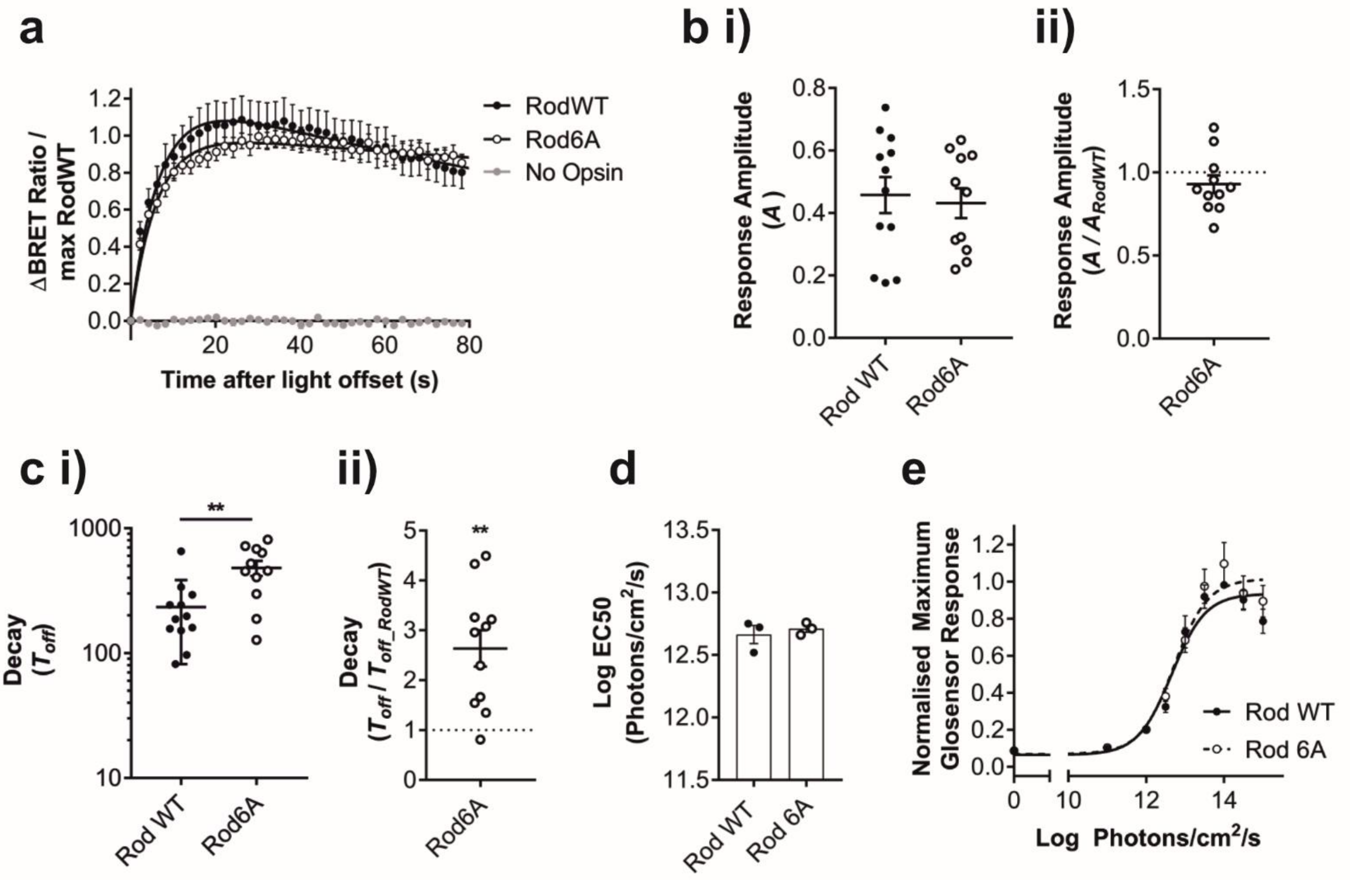

Supplementary Figure 2.

a) Time course of BRET response to 1s 485nm light (16.5 log photons). Data are normalised to pre-flash baseline (=0) and maximum response of rod opsin (=1). **b)** Response amplitude, measured as best fit scaling factor (*A*) normalised to RodWT, is similar between Rod6A and RodWT, two-tailed Mann-Whitney U-test, p = 0.694.**c)** Response decay measured as best fit T_{off} (s) is slower in Rod6A compared to RodWT, two-tailed Mann-Whitney U-test, p = 0.007.**d)** Sensitivity (measured as Log EC50, log photons/cm^2/s using Glosensor Gso assay) is comparable between Rod6A and RodWT.**e)** Irradiance response curves (IRC) fit to RodWT or Rod6A-driven responses to increasing intensities of 470nm light (11-15log photons) measured using Glosensor Gso assay. Data are mean ± standard error of mean. BRET data are mean of n = 11-12 replicates from 4 separate transfections. Glo Gso data are mean of 3 independent experiments. ** p > 0.01, p > 0.05 where no asterisk is displayed.

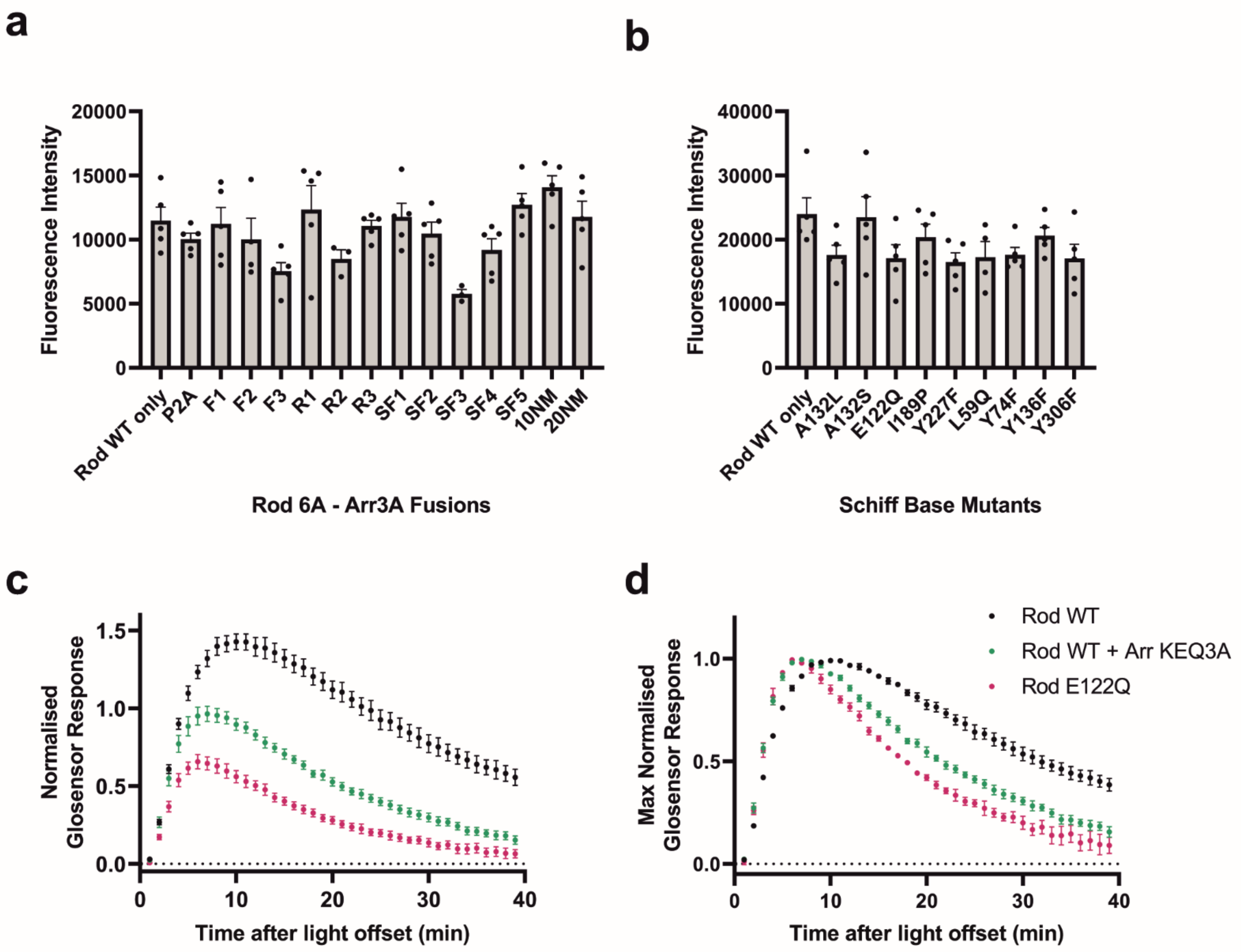

Supplementary Figure 3.

To assess fluorescence intensity, as a measure of expression level, we calculated the average fluorescence intensity for randomly selected cells from 40x magnification images of Hek293 cells transfected with **a)** Rod6A-Arrestin3A fusions (stained with 4D2 anti-rod opsin) or **b)** Schiff base mutants (stained with 1D4 anti-rod opsin). **c-d)** To confirm that both co-expression with arrestin and Schiff base mutants could decrease secondary messenger profiles, we examined response to light flash (15.6 log photons/cm^2/s) for RodWT, Rod E122Q and Rod WT co-transfected with ArrKEQ3A using the Glosensor Gso assay (n = 4 replicates per group) Responses in **c)** were normalised by dividing each raw luminescence data point by the last baseline luminescence value before stimulus and then **d)** normalised to maximum value to confirm these interventions improve off kinetics, and do not merely reduce response amplitude.

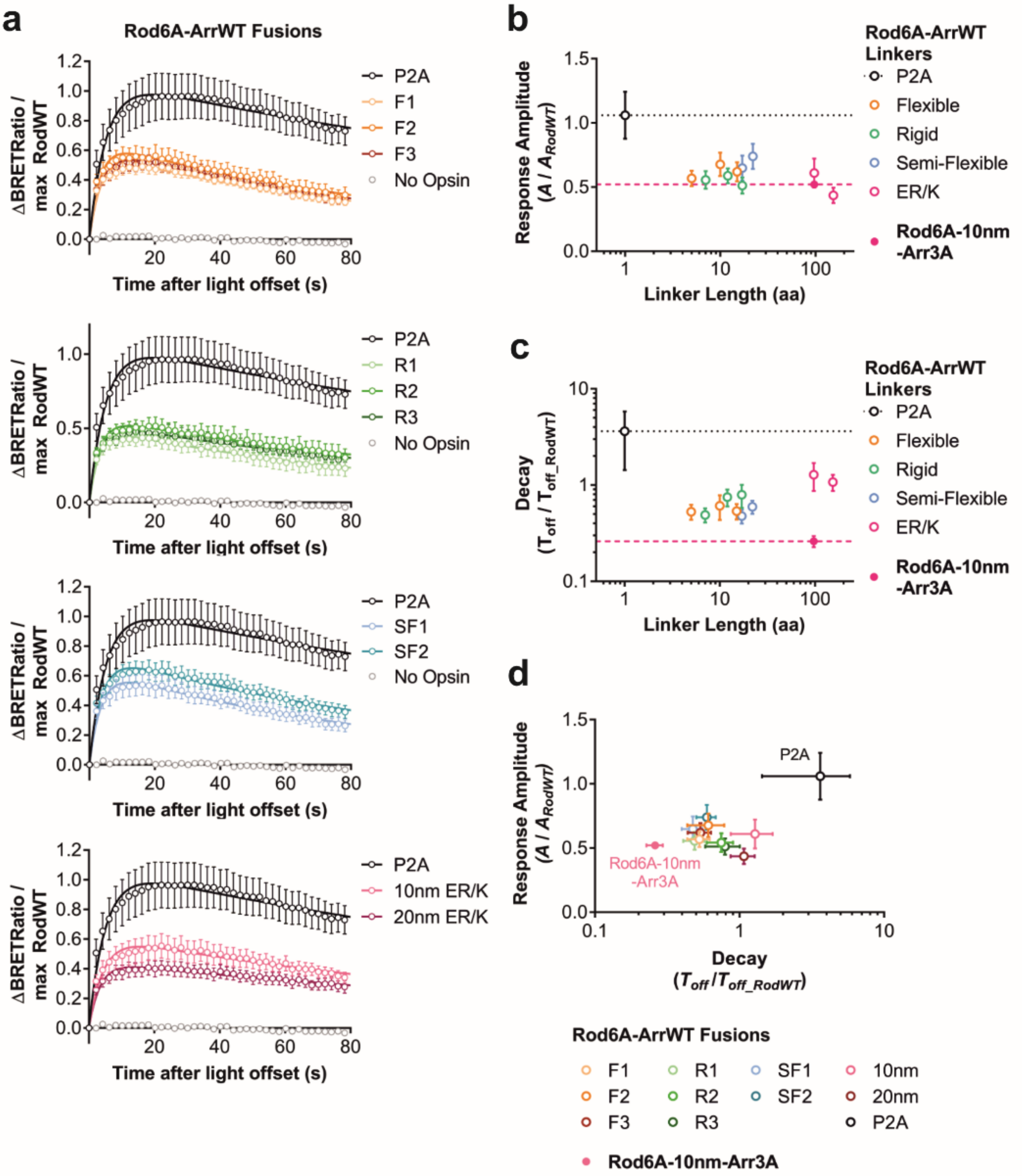

Supplementary Figure 4.

a) Time course of BRET light responses for co-expression or fusions of phosphonull rod opsin mutant (Rod6A) and wildtype arrestin (ArrWT). Responses are to 1s 485nm light (16.5 log photons). Data are normalised to pre-flash baseline (=0) and maximum response of rod opsin positive control (=1). **b)** Response amplitude (measured as fold change in best fit scaling factor *A* from Rod opsin positive control) is decreased for fusion constructs compared to co-expression with bicistronic P2A vector (dashed line), with no obvious relationship of linker length or composition with amplitude. Response amplitude for most fusions is comparable to best-performing Rod6A-Arr3A fusion with 10nm linker (dotted line). **c)** Response decay (measured as fold change in best fit T_{off} ,s from Rod opsin positive control) is faster for all fusion constructs compared to co-expression with P2A (dashed line). Response decay for R6-ArrWT fusions is slower than for best performing Rod6A-10nm-Arr3A fusion (dotted line) **d)** Comparing response amplitude and decay (measured as scaling factor *A* and T_{off} , respectively) of Rod6A-ArrWT fusions show all have slower decay and similar response amplitude to best-performing Rod6A-Arr3A fusion, Rod6A-10nm-Arr3A. Data shown are mean ± standard deviation of n = 12-13 replicates for Rod6A-ArrWT fusions and n = 17 for RodWT from 4 separate transfections.

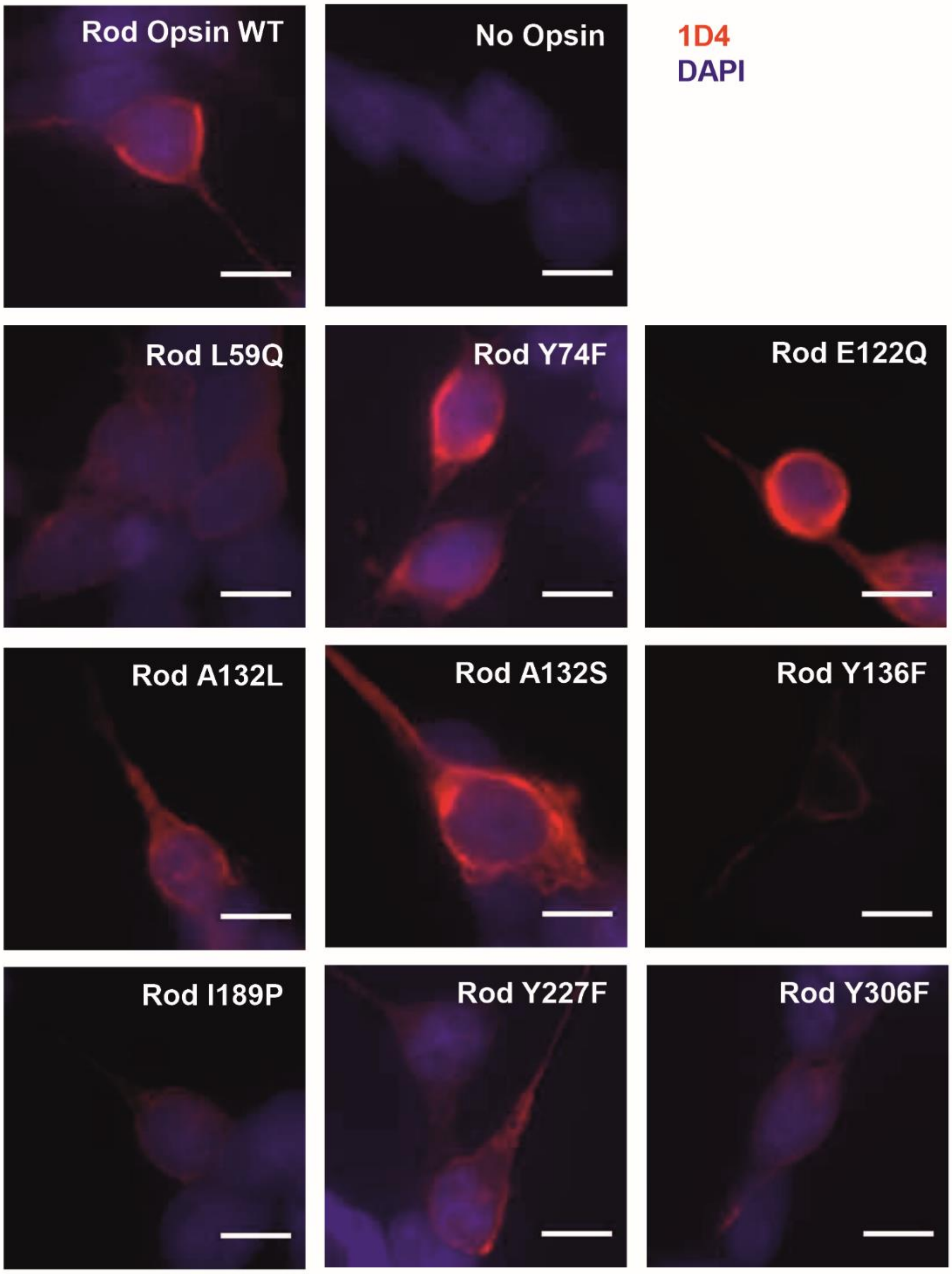

Supplementary Figure 5.

Heterologous expression of wildtype Rod opsin (RWT) or rod opsin meta-II decay mutants in HEK293T cells labelled with anti-rhodopsin 1D4 antibody (red) and DAPI nuclear stain (blue). Scale bar = 10μm.

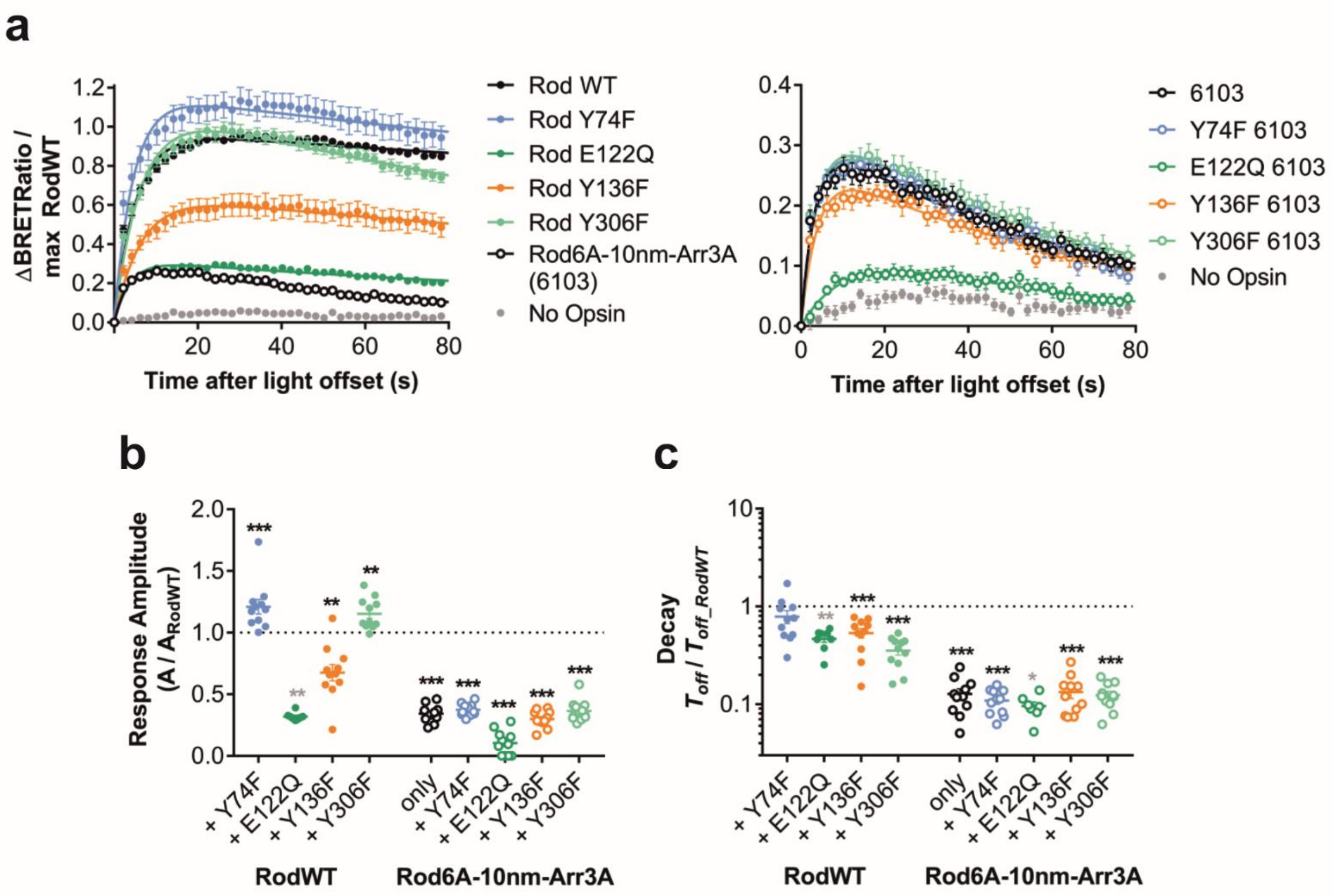

Supplementary Figure 6.

a) Time course of BRET light responses to 1s 485nm light (16.5 log photons). Data are normalised to pre-flash baseline (=0) and maximum response of rod opsin control (=1). Meta-II decay mutants applied to RodWT are shown as filled circles, while meta-II decay mutants applied to Rod6A-10nm-Arr3A fusion (6103) are shown as unfilled circles. **b)** Response amplitude (measured as fold change in best fit scaling factor *A* from Rod opsin positive control) is significantly attenuated for all Rod6A-10nm-Arr3A fusion with meta-II decay mutants, and is comparable with relative amplitude of Rod6A-10nm-Arr3A without meta-II decay mutation. We were unable to accurately fit model to 4/11 replicates for E122Q R6-10nm-Arr3A, missing values are given response amplitude value of zero to avoid biasing average towards replicates with larger amplitudes. **c)** Response decay (measured as fold change in best fit T_{off} ,s from Rod opsin positive control) is also comparable for all Rod6A-10nm-Arr3A fusions with meta-II decay mutants. Relative Toff is shown for E122Q Rod6A-10nm-Arr3A responses that could be fit using 3 parameter model (7/11 replicates). Data shown are mean ± standard error of mean of n = 8-12 replicates from 3 separate transfections. In **b-c)** a two-tailed one-sample Wilcoxon signed ranks test was used to compare each condition relative to Rod control (Theoretical median = 1). * p < 0.05, ** p < 0.01; *** p < 0.001, not significant where no asterisk is displayed. Grey asterisk = significant for uncorrected alpha (0.05), Black asterisk = significant for Sidak corrected alpha (0.006 for *A* and T_{off}).
